# The evolution of the Galápagos mantle plume

**DOI:** 10.1126/sciadv.add5030

**Published:** 2023-03-10

**Authors:** Caroline R. Soderman, Oliver Shorttle, Esteban Gazel, Dennis J. Geist, Simon Matthews, Helen M. Williams

**Affiliations:** ^1^ Department of Earth Sciences, University of Cambridge, Cambridge, UK.; ^2^ Institute of Astronomy, University of Cambridge, Cambridge, UK.; ^3^ Department of Earth and Atmospheric Sciences, Cornell University, Ithaca, NY, USA.; ^4^ Department of Geological Sciences, University of Idaho, Moscow, ID, USA.; ^5^ Institute of Earth Sciences, University of Iceland, Reykjavík, Iceland.

## Abstract

The lavas associated with mantle plumes may sample domains throughout Earth’s mantle and probe its dynamics. However, plume studies are often only able to take snapshots in time, usually of the most recent plume activity, leaving the chemical and geodynamic evolution of major convective upwellings in Earth’s mantle poorly constrained. Here, we report the geodynamically key information of how the lithology and density of a plume change from plume head phase to tail. We use iron stable isotopes and thermodynamic modeling to show that the Galápagos plume has contained small, nearly constant, amounts of dense recycled crust over its 90-million-year history. Despite a temporal evolution in the amount of recycled crust-derived melt in Galápagos-related lavas, we show that this can be explained by plume cooling alone, without associated changes in the plume’s mantle source; results are also consistent with a plume rooted in a lower mantle low-velocity zone also sampling primordial components.

## Teaser

The Galápagos plume has contained small amounts of pyroxenite over its history, decreasing its buoyancy through time.

## INTRODUCTION

The nature and temporal evolution of mantle material sampled by hot upwellings (plumes) is enigmatic. Many mantle plumes are thought to carry lower mantle material, which may be associated with large low–shear velocity provinces (LLSVPs) present at the core-mantle boundary (*1*, *2*). This lower mantle material may be primordial, as identified by W, He, and Ne isotopes [e.g., (*3*–*5*)], and/or may be related to the remains of dense subducted oceanic crust recycled back into the mantle by tectonic processes (*6*–*9*). The entrained material is carried into the upper mantle, where it is sampled in the melt source regions of some ocean island basalts (OIBs) erupted from mantle plumes, such as those found in Galápagos. Some heterogeneous material sampled by OIBs may also originate in the upper mantle directly, such as that sampled by the Ascension, Cape Verde, and Cameroon plumes, which may not have deep mantle roots (*10*). Therefore, mantle plumes and their associated erupted products provide a window into the nature of Earth’s heterogeneous mantle at different depths and length scales.

Plumes typically progress from a hot plume head stage to a subsequent narrower, cooler, conduit (“tail”), which produces steady-state OIB volcanism (*11*). This well-documented plume cooling (*12*, *13*) should result in a decrease in a plume’s ability to carry dense material, such as recycled crust (present as eclogite and pyroxenite mantle lithologies in the mantle) (*14*), as plumes’ elevated temperatures compensate for the compositional negative buoyancy arising from entrained dense lithologies. However, this basic chemical-geodynamic prediction is rarely able to be tested, as the evolution of plume lithology is often not documented or preserved in the geological record. Most geochemical studies of plume lithology take snapshots in time (usually, the modern-day expression of a plume), providing estimates of up to 20% pyroxenite or eclogite in the Iceland, Hawai’i, and modern Galápagos plumes, which could contribute up to 100% of melt depending on the melting conditions [e.g., (*15*–*18*)]. However, such studies miss the geodynamically critical information on how plume lithology, and therefore buoyancy, evolves throughout plume evolution. Geodynamic studies that include plume evolution generally do not support large fractions of pyroxenite being entrained by mantle plumes, because recycled crust is dense; thus, it should only constitute <10% of buoyant plumes (*19*–*21*). A challenge is therefore how to trace and quantify the presence of small mass fractions of pyroxenite in mantle source regions through a plume’s history. Ideal tools for this problem are geochemical tracers that are sensitive to both pyroxenite and more depleted lithologies in a plume: These tracers complement traditional proxies of pyroxenite melting that use incompatible trace elements and radiogenic isotopes, the budgets of which in lavas may be dominated by even small amounts of pyroxenite involvement in melt generation.

Stable Fe isotopes in basalts trace mineralogical heterogeneity in their mantle source through their sensitivity to recycled crust (*22*). Modeling and studies of natural samples have shown that both mantle temperature and lithological heterogeneity in a mantle source could be reflected in the Fe isotope composition of its partial melts (*18*, *22*–*24*), where the isotope composition is reported as δ^57^Fe, per mil deviation in ^57^Fe/^54^Fe relative to the IRMM-014 standard. Because the Fe abundances of melts derived from pyroxenite and peridotite are similar, the δ^57^Fe of mantle-derived melt reflects the relative contributions of the pyroxenite and peridotite to the bulk melt (*25*). Mantle pyroxenites are proposed to have a heavier Fe isotope composition (higher δ^57^Fe) than mantle peridotite, either because of heavier [mid-ocean ridge basalt (MORB)–like] bulk Fe isotope composition than peridotite (*24*) or because of the role of residual garnet during pyroxenite melting (*21*) and increased isotopically heavy pyroxene in the source compared to peridotite (*25*). Therefore, high δ^57^Fe has been used as an indication of mantle pyroxenite in several OIBs (*23*, *26*), including for the plume-influenced Galápagos Spreading Center (*18*).

The Galápagos mantle plume presents an outstanding opportunity to study plume evolution because all stages of its 90–million year (Ma) history are recorded in erupted basalts either in the Pacific, in terranes accreted onto Central America (*27*, *28*), or in the modern Galápagos (the present-day products of the plume tail). The Galápagos plume is thought to have cooled by up to 400°C since the plume head stage, with a maximum mantle potential temperature (*T*_p_) of 1700° to 1800°C proposed in the Tortugal suite (Costa Rica) and present-day plume tail estimates of *T*_p_ = 1360° to 1490°C (*12*, *13*, *29*–*31*). Geochemically distinct mantle components similar to those measured in the modern Galápagos have been identified in the plume head stage using radiogenic isotopes (*32*, *33*), although trace elements in olivine only show a recycled crustal component after the plume head (*13*, *33*), and multiple studies suggest that pyroxenite is present in the modern plume (*16*, *18*, *32*, *34*). The geochemical and lithological heterogeneity in the modern Galápagos has been linked to recycled crust stored in the lower mantle, within or near the Pacific LLSVP (*3*, *34*, *35*). In addition, a compositionally distinct ultralow-velocity zone (ULVZ) on the LLSVP margin has been associated with the plume and may also contain recycled crust (*36*). A primordial mantle component potentially located in the lower mantle or near the core-mantle boundary, recorded by high ^3^He/^4^He and near-solar Ne isotopic compositions in basalts erupted in Fernandina (*37*), is also proposed to be present in the modern Galápagos and by inference in the LLSVP (*3*). However, because pyroxenite is enriched in most geochemical tracers relative to peridotite and pyroxenite typically melts at lower temperatures and more productively than peridotite, most traditional geochemical tracers are dominated by contributions from pyroxenite melts, which complicates the relationship between melt composition and mantle composition. Therefore, it is unclear whether pyroxenite has been present throughout the Galápagos plume’s history, having been diluted by higher degrees of melting in the hotter plume head stage, or only appeared after some amount of plume cooling, despite the unfavorable geodynamical implications of such an increase in plume density as it cools (*33*).

To address this problem, we measured δ^57^Fe of a suite of well-characterized basalts and picrites from three periods in the evolution of the Galápagos plume, from the approximately 70- to 90-Ma plume head [Tortugal, Curaçao (Lesser Antilles), and Gorgona Island (Colombia)], 60- to 70-Ma head-tail transitional accreted terranes [Quepos (Costa Rica) and Azuero Peninsula (Panama)], and modern (<2 Ma) steady-state plume (Galápagos; fig. S1, data S1, and Materials and Methods). We analyzed samples from five southwestern and western Galápagos volcanoes, which span multiple isotopic domains of the recent archipelago (*38*), including samples from Fernandina volcano, which are proposed to overlie the current plume location and where the most extreme signatures of primordial mantle have been recorded in the plume’s history (*5*, *37*). Iron separation and isotope measurements were performed at the Department of Earth Sciences, University of Cambridge following established procedures (Materials and Methods). Measurements were made on a Neptune Plus multicollector inductively coupled plasma mass spectrometer (MC-ICPMS) in wet plasma, with typical 2 SEs on multiple δ^57/54^Fe measurements of the same sample better than 0.02‰ and measurements of reference materials in agreement with accepted values (data S2).

## RESULTS

**Fig. 1.**
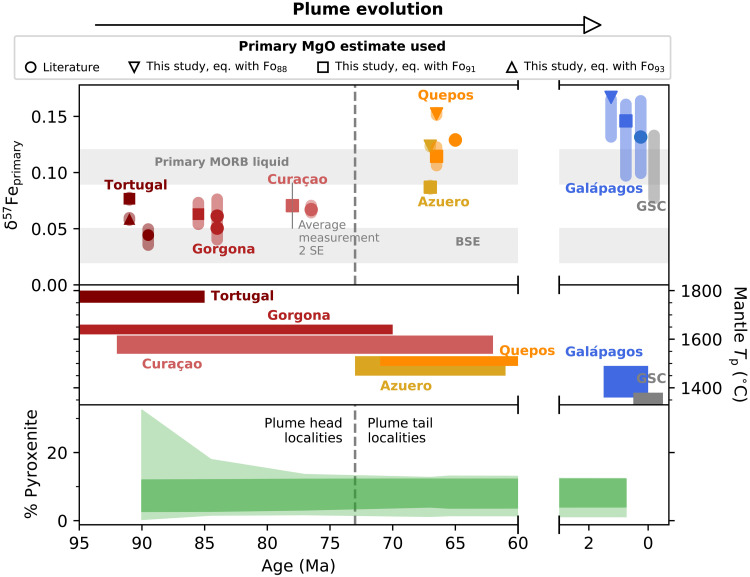
Primary δ^57^Fe throughout Galápagos plume evolution. The correction to δ^57^Fe_primary_ from the measured δ^57^Fe depends on how the primary liquid MgO is calculated (Supplementary Text). The uncertainty on each point reflects both the range of primary MgO estimates, and the resulting uncertainty on δ^57^Fe_primary_ (which depends on the slope of the δ^57^Fe─MgO fit), with the typical analytical error shown separately. GSC, Galápagos Spreading Center; raw data from (*18*). See Supplementary Text for sources of bulk silicate Earth (BSE) isotope composition. Primary MORB liquids from (*24*, *39*). Published mantle potential temperature (*T*_p_) estimates are mostly calculated from major element chemistry (*12*, *13*, *30*, *33*), and we use the middle panel to show the range of ages proposed for each locality (data sources for both age and temperature are given in table S2). We note that a consideration of harzburgite in the mantle source allows lower *T*_p_ estimates (*31*), but these estimates do not exist for all localities studied here. The bottom panel shows the calculated evolution of the pyroxenite fraction (5th and 95th percentiles of modelled solutions), with the minimum misfit solution in bold (see also Fig. 2). For the top and bottom panels, the data are plotted at the average age of the range shown in the middle panel.

Because fractional crystallization produces δ^57^Fe_measured_ that are different from δ^57^Fe_primary_ (the δ^57^Fe of the melt before crystallization), we correct our data back to an estimated primary liquid composition (Supplementary Text and data S3). As the plume ages and cools, δ^57^Fe_primary_ is observed to increase from plume head to tail (Fig. 1). Figure 1 also shows published Galápagos Spreading Center samples for comparison (*18*), pooled to represent an average melt sampled at the ridge (Supplementary Text). The modern Galápagos samples have been combined into one dataset, because all but one of the studied volcanoes are proposed to originate from a relatively uniform source lithology (*34*), but intervolcano variability is discussed in Supplementary Text.

### Peridotite melting cannot generate observed δ^57^Fe

**Fig. 2.**
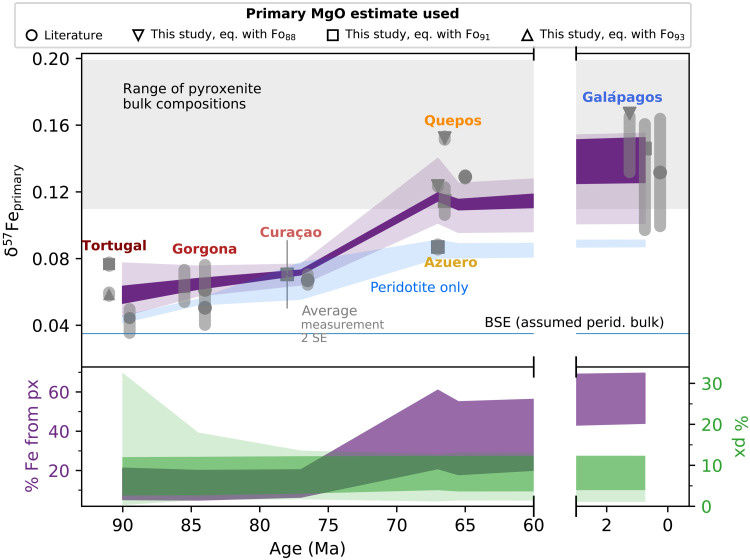
Minimum misfit δ^57^Fe_primary_ of the aggregate multilithology melt and pyroxenite fraction in the source from the Monte Carlo simulation. The bold bars show the range of best-fit results for the range of bulk pyroxenite isotope compositions considered (δ^57^Fe in purple, top; pyroxenite fraction in green, bottom). The paler envelopes show the 5th and 95th percentiles of the pyroxenite fractions (green, bottom) and resulting δ^57^Fe_primary_ (purple, top) from the Monte Carlo runs accepted at 95% confidence, compared to the data and the peridotite-only (blue) case. The bottom panel also shows, in purple, the fraction of Fe in the aggregate melt derived from pyroxenite. The pyroxenite fraction results are repeated from Fig. 1 to highlight the balance and distinction between changes in fraction of pyroxenite, and fraction of Fe derived from pyroxenite, as the plume evolves.

The increase in δ^57^Fe_primary_ from plume head to present day coincides with ∼ 400°C of plume cooling (1800° to 1400°C) (*12*, *29*, *30*). Smaller extents of melting from a cooler plume increase ∆^57^Fe_melt−source_ and create heavier Fe isotopic compositions in the melt (*22*, *40*). However, when we test this null hypothesis using our self-consistent mantle melting and equilibrium isotope fractionation model (*22*, *24*), we find that cooling of a peridotite-only lithology cannot match the observations (Fig. 2, blue shading). The modeled increase in δ^57^Fe flattens for *T*_p_ below 1500°C (because the maximum mantle-melt isotopic fractionation is approached), whereas the data suggest a continuing increase in δ^57^Fe_primary_ from Azuero and Quepos (60 to 70 Ma) to modern Galápagos (present day). The gradual increase in δ^57^Fe as the plume cools in this model is driven both by decreasing average melt fraction and by increasing Fe^3+^/Fe_T_ of the melt, as the average pressure of melting decreases with decreasing *T*_p_ (*22*, *41*). We note that our model of peridotite melting can reproduce the calculated primary MORB liquid (*39*) and Galápagos Spreading Center δ^57^Fe (fig. S4), although a contribution from pyroxenite melting is required for the isotopically heaviest Galápagos Spreading Center samples (*18*).

### Evidence for a pyroxenite component in the plume

Given the poor fit of pure peridotite melting models to the data, we next test the hypothesis of a pyroxenite component in the cooling plume. Because pyroxenite lithologies are likely to have higher δ^57^Fe than peridotite (largely because they inherit a high δ^57^Fe from their MORB protolith) (*23*, *25*), the presence of pyroxenite in the plume is expected to produce higher δ^57^Fe melts than for pure peridotite melting. As pyroxenite is more fusible than peridotite, we also expect the contribution from pyroxenite-derived melts to increase as the plume cools. To model the isotopic effect of pyroxenite, we have used the melting behavior and major element composition of an average of global pyroxenite lithologies observed from the mantle (MIX1G; Supplementary Text). We consider a range of bulk δ^57^Fe values for the pyroxenite (0.11, 0.15, and 0.20‰), which span the range of average MORB data for each mid-ocean ridge segment given the likely origin of mantle pyroxenite as recycled crust and includes the δ^57^Fe = 0.15‰ proposed as average MORB (Supplementary Text) (*39*). This range in pyroxenite δ^57^Fe also allows for a consideration that the bulk isotope composition of a mantle pyroxenite may be lower than average global MORB (0.15‰) because of its possible formation by hybridization with mantle peridotite [e.g., (*15*)]. With a Monte Carlo simulation, we explore the aggregate melt δ^57^Fe (a mixture of peridotite and pyroxenite melt) for a range of pyroxenite fractions and mantle temperatures allowed for a buoyant and progressively cooling plume (Supplementary Text). For each locality, the simulation calculates the Fe isotope composition of a mixture of peridotite and pyroxenite-derived melts, considering the pyroxenite fraction, temperature, and melting behaviors and melt compositions of each lithology. This modeling shows that, for the bulk pyroxenite isotope compositions considered, the observed Fe isotope evolution of the plume is best matched by a small and approximately constant pyroxenite fraction, <12%, in the plume before it has undergone melting (Figs. 1 and 2), consistent throughout both plume head and tail.

At high mantle *T*_p_ in the plume head, a wide range in pyroxenite fraction could be consistent with the natural data, as shown by the 5th and 95th percentiles of accepted model solutions in Figs. 1 and 2. This result occurs because the Fe fraction in the bulk melt contributed from pyroxenite is small in the plume head relative to that in the plume tail, because peridotite is melting to relatively high degrees in the plume head, and the Fe-isotopic fractionation of melts relative to their sources is small at high temperature. Therefore, although the best-fit model solutions suggest <12% pyroxenite present in the mantle source of Tortugal, for example, a good fit (within 95th percentile of solutions) to the Fe isotope data could still be achieved with up to 30% source pyroxenite, depending on the bulk isotope composition of the pyroxenite. Olivine trace element data for Tortugal (see Supplementary Text and fig. S10) are variable but consistent with 10% pyroxenite fraction in the best-fit solutions.

As the *T*_p_ decreases, there is a decrease in the fraction of the total melt coming from peridotite compared to pyroxenite because of the more fusible nature of pyroxenite relative to peridotite. This decrease is manifested in a corresponding increase in the proportion of Fe in the bulk melt originating from pyroxenite, which has a marked influence on the δ^57^Fe budget of the bulk melt. This increased contribution of pyroxenite components to melt Fe budget means that younger melts generated from a cooler plume will have δ^57^Fe_primary_ values that become progressively dominated by the pyroxenite component in their source region. The net result is a steeper increase in melt δ^57^Fe_primary_ with decreasing plume age compared to that expected for a pure peridotite source.

Our results therefore support the hypothesis of a pyroxenite component present throughout the history of the Galápagos plume (*13*, *33*), but, critically, we show that plume cooling alone can generate the increase in the amount of pyroxenite-derived melt, without relying on any ad hoc and potentially geodynamically unlikely scenarios that involve increasing the amount of pyroxenite in the source with time (*33*). Critically, as the plume cools through time, the fraction of Fe being derived from peridotite melt falls, and the δ^57^Fe of the aggregate peridotite and pyroxenite melt increases because of the increased contribution of pyroxenite lithologies to melt Fe budget, even for a constant pyroxenite fraction in the mantle source. The presence of a pyroxenite component, and its proportion in the southwestern and western modern Galápagos, is consistent with published results for the present-day plume (*16*, *18*, *34*). For example, for most of the modern volcanoes studied here, Gleeson *et al.* (*34*) propose less than 5% pyroxenite in their source based on major element analysis, which is consistent with the lower range of our best-fit estimates based on Fe isotopes. The intervolcano variability in the modern Galápagos is discussed further in Supplementary Text (fig. S9).

As discussed above, the pyroxenite component used in our models was assigned values of bulk δ^57^Fe ranging from 0.11 to 0.20‰ (section S3), and there is a minor trade-off between pyroxenite composition and fraction in the plume. The proportion of pyroxenite required decreases as its bulk δ^57^Fe becomes higher, with approximately 12% pyroxenite for a bulk composition of 0.11‰ and 3 to 4% pyroxenite for a bulk composition of 0.20‰. If we use a relatively extreme bulk pyroxenite δ^57^Fe = 0.3‰ (*18*, *23*) [although, the processes required to form such a heavy pyroxenite remain enigmatic (*24*)], then the modeled trend of δ^57^Fe would become steeper and smaller pyroxenite fractions would reproduce the observed trend. However, critically, our overall conclusions of plume lithology evolution would be unchanged, given that all the best-fit solutions regardless of bulk pyroxenite isotope composition considered favor only small changes in pyroxenite fraction through time (table S3).

Olivine trace elements are also widely used as tracers of pyroxenite in the source region of basalts and have been taken to suggest a pyroxenite component in the Galápagos plume, although, with these tracers, the pyroxenite only becomes resolvable (hence, has been proposed to increase in proportion in the plume) after the plume head stage (*13*, *30*, *33*). We evaluate the olivine trace element results in the context of our model by estimating the equilibrium olivine trace element composition for the bilithologic plume using composition-dependent olivine-melt partition coefficients (Supplementary Text and table S5). We find that an approximately constant pyroxenite fraction through plume history is also consistent with the olivine trace element contents (fig. S10), with our modeled olivine trace elements generally reproducing trends in the natural olivine data. An increase in pyroxenite fraction through time is therefore not required to generate the trace element trends seen in natural data throughout plume evolution, with a cooling plume also able to generate variations in olivine trace element compositions consistent with natural data. However, the higher temperatures of the plume head than the modern Galápagos mean that pyroxenite-derived melts are diluted more by peridotite-derived melts in the plume head than the plume tail, allowing high pyroxenite fractions to be consistent with observed geochemistry: This is true for both Fe isotopes and olivine compositions.

These results show that, when pyroxenite fractions are small, as modeled here, modeled Fe stable isotopes may be a more discriminating tracer of mantle source lithology than modeled trace element abundances in olivine. Modeled olivines derived from peridotite and bilithologic mantle melts have similar compositions for much of the temperature range considered throughout the plume’s history (fig. S10), largely because, at high temperature, the olivines are dominated by contributions from peridotite across the range of small plausible pyroxenite fractions.

## DISCUSSION

### Buoyancy constraints on the fraction and density of entrained lower mantle material

Large pyroxenite fractions will produce a negatively buoyant plume because of their excess density relative to ambient mantle (*7*): A mantle *T*_p_ of ∼1430°C is required for a plume with 5% recycled crust to be neutrally buoyant (not even actively upwelling) in the upper mantle (*10*). Hence, geodynamical modeling predicts that only small fractions of dense recycled crust should be entrained by an upwelling plume (*14*, *21*). Our results are clear geochemical evidence supporting this geodynamic behavior. In our best-fit model solutions for each of the three bulk pyroxenite isotope compositions considered, the Galápagos plume buoyancy [calculated following (*17*, *42*); see Supplementary Text] decreases over time, but the plume remains positively buoyant right up to the modern day (table S3). This inferred positive buoyancy of the plume is consistent with the ability of the Galápagos plume to also carry deep primordial mantle material [potentially a denser component than ambient mantle (*43*)] throughout its lifetime, even now at the coolest point in its history. We note that the solution for the lowest pyroxenite δ^57^Fe_bulk_ considered, 0.11‰, produces a modern plume only just buoyant (excess density of −1 kg/m^3^; table S3). This result advocates for the lower limit for the bulk isotope composition of the pyroxenite component to be close to 0.11‰; for any lighter bulk compositions, the proportion of pyroxenite required would produce a negatively buoyant plume.

**Fig. 3.**
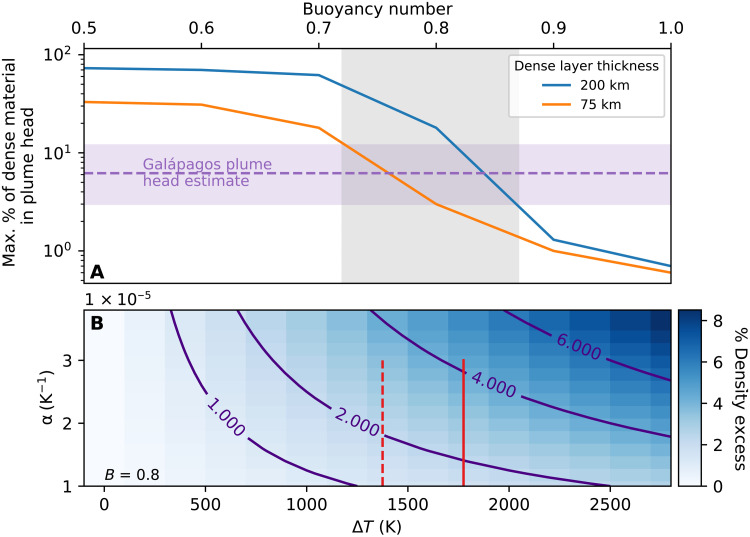
Constraints from pyroxenite entrainment fraction on the density of entrained material. (**A**) Modified from (*14*), estimates of maximum entrainment fraction of dense material in the plume head for different boundary layer thicknesses of that material. The grey bar shows the buoyancy number implied by the range of minimum misfit pyroxenite fractions in Tortugal for the range of bulk pyroxenite isotope compositions considered (purple shading). Buoyancy number, *B*, is defined as ∆ρ/(α ∆*T* ρ_c_); Supplementary Text. (**B**) Estimate of excess density (red lines) of entrained material in the Galápagos plume relative to ambient mantle, based on *B* = 0.8 and using suitable estimates of thermal expansion, α and driving temperature contrast, ∆*T* = *T*_CMB_ − *T*_p_ (see section S7). The contours mark density excesses in α-∆*T* space, as shown by the color bar. The solid red line shows density results using the ∆*T* estimate using the Tortugal *T*_p_, and the red dashed line shows results using the ∆*T* estimate considering non-adiabatic cooling on the lower mantle *T*_p_ of Tortugal (section S7).

Geodynamic models also show that the entrainment of dense mantle material (e.g., recycled crust) by a plume is dependent on the density contrast between the dense component and the surrounding mantle (*14*, *19*). Here, we use our calculated pyroxenite fraction to place constraints on the density of the material being carried by the plume (Fig. 3), were it to be carried from the lower mantle, such as from near to or within an LLSVP, including the ULVZ on the LLSVP associated with the plume (*36*). First, without considering the presence of an ULVZ [which affects entrainment dynamics (*14*)], our estimate of maximum pyroxenite fraction entrained in the plume head constrains the buoyancy number of the plume [ratio of chemical density contrast of dense material to the thermal density contrast of the hot thermal boundary layer, following (*14*); Supplementary Text] to be ~0.7 to 0.9 (Fig. 3A), consistent with estimates used in existing geodynamic models (*21*). Given the estimates of thermal expansion and the temperature contrast (Δ*T*) between the core-mantle boundary and the potential temperature of the mantle that drives the upwelling plume (as described in section S7) and using the average buoyancy number of 0.8, we estimate that the pyroxenite component has an excess density of 1.1 to 4.3% relative to ambient mantle (red lines in Fig. 3B). This excess density is in agreement with the predicted excess density of MORB stored in the lower mantle and of LLSVPs (*6*, *7*, *44*).

Models have also suggested that relatively high proportions (e.g., >1%) of entrained pyroxenite are maintained over more of a plume’s history if ultradense material is also present in the lower mantle, compared to a case when no ultra-dense material is present (*14*). In this context, our finding of a sustained presence of pyroxenite in the Galápagos plume until the present day supports geophysical evidence for the plume being rooted in an ULVZ (*36*).

Our results therefore provide important constraints on the composition and entrainment of material stored in or around LLSVPs by mantle plumes, given the Galápagos plume’s proximity to the Pacific LLSVP and ULVZ. We have clear evidence for a pyroxenite lithology entrained into the mantle plume source. Given the low solidus temperature of pyroxenite relative to peridotite, pyroxenite should contribute progressively more melt as the plume temperature decreases, and the isotopically heavy component in the Galápagos plume behaves consistently with that prediction. If this isotopically heavy pyroxenite is sourced from the lower mantle, then either the Pacific LLSVP is itself a pile of recycled crust (*6*–*9*) or dense recycled crust can be mixed into, or extracted from near to, the LLSVP (*8*, *21*).

### Implications for our understanding of primordial geochemical signatures

The lower mantle may also contain primordial material, which can be entrained in small proportions by an upwelling plume (*3*, *20*, *21*). Modern Galápagos shows the most negative μ^182^W anomalies yet measured in OIB (*5*), consistent with a primordial lower mantle/core component entrained in the plume, despite recycled crustal material being able to overprint primordial W (and He) isotope signatures (*4*). We have included Fe isotope data from the Fernandina volcano in our study, the proposed current Galápagos plume location, where μ^182^W of −22 and ^3^He/^4^He of 30 *R*/*R*_A_ (where *R*_A _is the present-day atmospheric ratio) have been reported (*5*, *37*). A small pyroxenite component would therefore be consistent with minimal W and He overprinting of primordial material by W- and ^4^He-rich crust and would support that both recycled and primordial material can be entrained by a plume, perhaps both from the lower mantle. The Fernandina samples themselves record δ^57^Fe_primary_ of ≥0.06‰ (dependent on ∆^57^Fe_olivine−melt_ used), isotopically lighter than the overall modern plume (Fig. 1; and shown compared to the other studied volcanoes in fig. S9). Although the modern Galápagos plume as a whole may have a 4 to 12% pyroxenite component, individual volcanoes will record subtly different components/geochemical domains, as has been well-recorded in Galápagos OIB (*3*, *32*, *38*). It is thus possible that Fernandina records a particularly small pyroxenite fraction, producing the δ^57^Fe most typical of peridotite melting at plume temperatures and, critically, with minimal overprinting of lower mantle primordial W and He isotope signatures. We note that samples with the most extreme μ^182^W (≥−17) and ^3^He/^4^He (≥25 *R*/*R*_A_) recorded in Samoa [Ofu (*5*, *45*)] also have lighter-than-MORB δ^57^Fe (δ^57^Fe_primary_ ≥ 0.07‰) compared to other samples from different Samoan volcanoes (*24*); Samoa’s Ofu volcano may similarly be sampling relatively less recycled crustal material than the other volcanoes in the Samoan plumbing system. More work needs to be done to confirm the tentative link seen here between isotopically light Fe isotope compositions and primordial geochemical signatures.

The variation of pyroxenite-derived melt throughout plume evolution also suggests that geochemical signatures of primordial mantle, which are used to investigate early Earth processes and mantle evolution, may be variably diluted by recycled crustal components throughout plume history (*4*). To explore this further, we have calculated the amount of pyroxenite-derived W in the aggregate melts for our minimum misfit model for each bulk pyroxenite isotope composition considered. We find that the proportion of pyroxenite-derived W is, as expected, smallest in the scenarios that predict the smallest pyroxenite fraction, but that for any given best-fit scenario, the fraction of W derived from pyroxenite through time only varies by 10 to 20% (table S3). Given the susceptibility of lower mantle μ^182^W and ^3^He/^4^He isotope signatures to overprinting by crustal material (*4*), we anticipate that the earlier plume head and tail localities may also show negative μ^182^W (and high ^3^He/^4^He), such as those observed in modern Galápagos samples. Confirmation of this hypothesis would provide important constraints on the dynamics of lower mantle evolution and its entrainment into the upper mantle, such as rates of mantle mixing and the extraction of volatiles from the deep Earth. Our work shows the potential of novel stable isotopes combined with self-consistent thermodynamic modeling to constrain mantle dynamics and plume evolution.

## MATERIALS AND METHODS

### Samples

The samples measured for δ^57^Fe in this study have been previously characterized for major and trace elements, with some samples also having radiogenic isotope and olivine trace element data. The data sources used are summarized in data S1, with a map (fig. S1) showing the geographic location of each locality studied. The raw δ^57^Fe data, including results for the standards, are in data S1 and S2.

### Fe separation and isotope analysis

Iron isotope analyses were carried out on dissolutions of 20 mg of whole-rock powders following established procedures (*46*, *47*) in two batches in 2018–2019 and 2021 at the University of Cambridge. Powders were dissolved in a ∼10:1 mixture of HF:HNO_3_ on a hot plate at 120°C for 48 hours, evaporated to dryness, and redissolved twice in 6 M HCl to remove fluorides. Each dissolution was brought up in 2 ml of 6 M HCl, and 0.5 ml was loaded onto AG1-X4 anion exchange resin, preconditioned with 1 ml of 6 M HCl. Matrix was eluted using 4 ml of 6 M HCl, and then iron was collected with 7 ml of 2 M HCl. A recalibration of the column chemistry in 2021 (after a laboratory shutdown because of coronavirus disease 2019 restrictions) resulted in requiring an extended Fe cut of 8 ml for samples run in 2021 (these samples are marked in data S1). The purified Fe solution was dried, refluxed in HNO_3_:H_2_O_2_, and dissolved in 4 M HNO_3_ before dilution to 0.1 M HNO_3_ for isotopic analysis.

Sample solutions were analyzed for Fe isotopes on a ThermoNeptune Plus MC-ICPMS at 6 parts per million (ppm) of Fe (2018–2019) or 8 ppm of Fe (2021–2022) in wet plasma and medium resolution mode. Samples were introduced using a quartz cyclonic spray chamber, and instrumental mass bias was corrected for by sample standard bracketing. Sample and standard iron beam intensities (typically 35 to 45 V of ^56^Fe on a 10^10^-ohm resistor) were matched to within 5%. Mass dependence, reproducibility, and accuracy were monitored by analysis of an in-house FeCl_3_ salt standard and international rock standards [Hawaiian Basalt (BHVO-2), Reykjavik Iceland Basalt (BIR-1), and Columbia River Basalt (BCR-2)] processed through column chemistry, giving values in agreement with previous studies (*48*). Repeat dissolutions and measurements in 2022 of samples and standards previously run in 2019 were identical, within error (data S1 and S2).

## Supplementary Materials

### This PDF file includes:

Supplementary Text Figs. S1 to S10 Tables S1 to S5 References
