# Supplementary material for: The evolution of the Galápagos mantle plume
Source: Sci Adv. 2023 Mar 10;9(10):eadd5030. doi: 10.1126/sciadv.add5030 (PMC10005182; doi:10.1126/sciadv.add5030)
Supplement: Supplementary file 1 — Supplementary Text Figs. S1 to S10 Tables S1 to S5 References [file sciadv.add5030_sm.pdf]

Science Advances
AAAS

Supplementary Materials for

The evolution of the Galápagos mantle plume

Caroline R. Soderman *et al.*

Corresponding author: Caroline R. Soderman, cs801@cam.ac.uk

Sci. Adv. **9**, eadd5030 (2023)
DOI: 10.1126/sciadv.add5030

The PDF file includes:

Supplementary Text
Figs. S1 to S10
Tables S1 to S5
References

Other Supplementary Material for this manuscript includes the following:

Data S1 to S3

1 Correction to primary δ^{57}Fe

In the samples studied here, δ^{57}Fe correlates with whole-rock MgO, as expected for fractional crystallisation and olivine accumulation trends *(49, 50)*. Low MgO samples from modern Galápagos, Azuero and Quepos show the highest δ^{57}Fe (0.16–0.21 ‰; MgO 5.6–11.1 wt%), and high MgO samples from Tortugal and Azuero show the lowest δ^{57}Fe (−0.05– 0.03 ‰; MgO 27.1–34.0 wt%). Olivine fractional crystallisation and/or accumulation can change the measured δ^{57}Fe from the primary liquid δ^{57}Fe. The correction for this process requires an estimate of primary liquid MgO, as well as an estimate of $\Delta^{57}Fe_{crystal-melt}$. While the latter can be theoretically calculated and estimated from natural samples, it is not generally well-constrained, with variable estimates that can have a large influence on the resulting primary δ^{57}Fe *(23, 24, 39)*. Therefore, given the wide MgO range of our samples for most localities and approximately linear δ^{57}Fe-MgO relationships, we construct an empirical fit in MgO-δ^{57}Fe space for each locality (Fig. S2), and take the primary δ^{57}Fe along that line depending on the proposed primary MgO. For Galápagos, where we do not have a wide MgO range of samples, we use the maximum and minimum slopes from the other localities, and note that the range of these slopes, at typical magmatic temperatures, approximates a $\Delta^{57}Fe_{crystal-melt}$ between -0.4 and $-0.1 \times 10^6/T^2$, consistent with literature estimates *(23, 24)*. The approach used for Tortugal, Gorgona, Curaçao, Quepos and Azuero assumes the samples at each locality represent a single liquid line of descent, which we consider to be a suitable approximation given the constant La/Sm ratio (where the data exists) for each locality (Fig. S3). Removing the one Azuero and one Tortugal sample which fall off the La/Sm trend for each locality from the δ^{57}Fe-MgO fitting results in no significant difference in the calculated primary δ^{57}Fe.

For the primary MgO, we take the results from a variety of methods (Table S1) to highlight the importance of primary MgO on the resulting primary δ^{57}Fe. For methods involving calculation of primary MgO, we used compiled published whole rock data for samples from each locality. For calculation in equilibrium with olivine of a given forsterite content, we added olivine back incrementally, calculating equilibrium liquid and olivine MgO at each step, until the desired forsterite content was reached. We used a Kd_{Fe-Mg} of 0.32 *(51)* and a Fe^{3+}/Fe_T of 0.156 (maximum for Galápagos of 0.175 *(52, 53)*, corrected by a factor of 1.125 following *(54)*), with only samples with MgO > 10 wt% used to avoid samples that may have crystallised pyroxene. For Tortugal, this filter was lowered to 8.5 % as no samples with higher MgO would solve for equilibrium melt compositions. The results of the primary δ^{57}Fe calculations are in Data S3.

The range of primary δ^{57}Fe shown in Fig. 1 for each approach to primary MgO calculation depends on both the range of primary MgO estimates, and the slope of the δ^{57}Fe-MgO fit in Fig. S2.

2 Calculation of pooled Galápagos Spreading Centre melts

δ^{57}Fe for the Galápagos Spreading Centre (GSC) has been published by *(18)*, for samples taken along an E-W transect across the (approximately) N-S spreading centre. Gleeson et al. *(18)* report variable δ^{57}Fe for D, N and

E-MORB samples, with a pyroxenite signature most apparent in E-MORB. To make a direct comparison between the spreading ridge samples (generally thought to be poorly mixed and homogenised in crustal magma chambers, (e.g., *(55)*) and the OIB settings, we calculate an aggregate GSC $\delta^{57}Fe_{primary}$ reflecting the contributions across the ridge. We correct the data from *(18)* for olivine fractional crystallisation following *(23, 24, 39)*, using a $\Delta^{57}Fe_{ol-melt}$ of $-0.4 \times 10^6/T^2$ and $\Delta^{57}Fe_{ol-melt}$ of $-0.1 \times 10^6/T^2$, which are upper and lower estimates *(23, 24)*. Samples are corrected back to equilibrium with Fo_{91} olivine using a K_d of 0.32 *(51)* and Fe^{3+}/Fe_T of 0.143 (average MORB, *(54)*). The aggregate melt $\delta^{57}Fe_{primary}$ is then calculated by mass balance, using the calculated $\delta^{57}Fe_{primary}$ and $FeO_{primary}$ for each sample, and the range shown in Fig. 1 represents the results for $\Delta^{57}Fe_{ol-melt} = -0.4$ and $-0.1 \times 10^6/T^2$.

We find that pure peridotite melting (with no lithospheric cap to the melting region, to represent the spreading centre) can explain the pooled GSC data, if $\Delta^{57}Fe_{ol-melt} = -0.4 \times 10^6/T^2$ is used (i.e., the isotopically lightest result shown by the grey bar in Fig. S4). A smaller $\Delta^{57}Fe_{ol-melt}$ would require contributions from an isotopically heavy pyroxenite-derived melt, as proposed by Gleeson et al. *(34)*.

3 Thermodynamic mantle melting and equilibrium isotope fractionation model

Our thermodynamic and isotope fractionation model combines modal mineralogy and phase chemistry information from calculations performed using the dataset of Holland et al. 2011 *(56)* and activity-composition (a-X) models of Holland et al. 2018 *(57)*, implemented in THERMOCALC *(58)*, with a calculation of composition- and temperature-dependent equilibrium isotopic composition of the phases present. Full details of the model are in Soderman et al. 2021, 2022 *(22, 24)*. The model results cover up to 40 kbar and 1100–1930 °C (KLB1) and 1100–1700 °C (MIX1G). We chose MIX1G, a silica-deficient pyroxenite composition, to represent a recycled crust-derived mantle component because it plots close to the average global pyroxenite composition *(59)*. For the bulk Fe isotope composition of the peridotite lithology, we use 0.035 ‰, as an average of the estimates given by *(39, 60)* which are shown in Fig. 1 of the main text. We also considered three different bulk pyroxenite isotope compositions, 0.11, 0.15 and 0.20 ‰. The intermediate value was chosen as the average MORB composition *(39)*, and the two extreme cases were chosen as the highest and lowest published average $\delta^{57}Fe$ amongst individual ridges in the global MORB dataset (MAR *(61, 62)*, and South China Sea *(63)* respectively); Fig. S5. This range therefore takes into account reasonable variation in the bulk isotope composition of recycled MORB. The lightest value may also be relevant for hybrid mantle pyroxenites, formed through reaction between eclogite and peridotite *(e.g., 15)*, and as discussed in Soderman et al. 2021 *(24)*. calculated complete isentropic decompression melting paths (i.e., no melt generated at higher pressures than our THERMOCALC results) for $T_p = 1300$ °C and $T_p = 1400$ °C (KLB1, MIX1G) and $T_p = 1530$ °C (KLB1); see *(22)*. Following Soderman et al. 2022 *(22)*, we then calculate an average pressure of melting along each of those isentropic decompression paths, with the only change from the calculations presented in *(22)* being the addition of a lithospheric cap on the melting region of 60 km. The

melt composition at the average pressure and temperature of melting is then used as an estimate of the aggregate melt composition (see *(22)*). Melt fraction and major element composition are calculated by THERMOCALC, with isotope composition calculated as described in *(22, 24)*. The lithospheric thickness used is based on the modern south-western Galápagos lithospheric thickness *(64)* and consistent with suggestions that the CLIP formed above relatively thick lithosphere (rather than excessively thinned due to stretching during the plume head stage; *(65)*). Although the lithospheric thickness at the time of eruption of plume head melts is less well-constrained than the modern plume, the results for the plume head are insensitive to lithospheric thickness due to the already high degree of melting.

We then approximate the average pressure of melting for all potential temperatures (not just those with calculated isentropic decompression melting paths) by assuming a linear interpolation and extrapolation between and beyond the calculated pressures, acknowledging that this approach likely results in a slight underestimation of average melting pressure at higher T_p. This underestimation occurs because the average pressure of melting is not predicted to be strictly linear with T_p, instead curving to deeper pressures at progressively higher T_p *(66)*. Using the interpolated average melting pressures and corresponding temperatures, we extract the melt composition and melt fraction at each point up to the P-T limits of our pseudosection, then parameterise the composition and melt fraction vs potential temperature behaviour, allowing us to extrapolate the melt composition and melt fraction to higher potential temperatures and pressures than covered in the pseudosection and applicable to our plume head localities. For this parameterisation, we use an additional high pressure and temperature constraint on the melt chemistry and melt fraction to ensure our model produces expected behaviour, particularly for the plume head localities. For KLB1, this high P-T constraint is taken from experimental melting results *(67)*. We estimate the expected melt fraction and composition from the experimental results for the average melting pressures calculated for a T_p of 1800 °C, the upper estimate for Tortugal, and use this as the extreme P-T fitting point. An example of this parameterisation is shown in Fig. S6. For MIX1G, we take the assumption of a linear T_{liquidus} - pressure behaviour *(66)* to predict where our extrapolated P_{average} results intersect the liquidus, giving us a P-T point where we expect complete melting (and therefore the melt composition to be that of the bulk system). The intersection is calculated to be at P = 63 kbar, T = 1954 °C, corresponding to a T_p of 1801 °C, and therefore we assume complete melting at this potential temperature to constrain the high P-T behaviour of MIX1G melts. At this potential temperature, the melt fraction of MIX1G is 1, and composition of the liquid is that of the bulk MIX1G system. An example of the parameterisation for melt fraction for MIX1G, combining the high P-T point and the THERMOCALC results, is shown in Fig. S6.

We note from the top panel of Fig. S6 that our linear interpolation approach to P_{average} compared to the non-linear solidus results in a slight decrease (by 0.018) of peridotite melt fraction with increasing T_p, up to 1450 °C. This is opposite behaviour to that expected for increasing temperature, however we do not consider that correcting this artefact of the model would affect our results. Since the peridotite melt-source isotope fractionations calculated for this low temperature range only vary by 0.002 ‰, this small decrease in melt fraction for $T_p < 1450$ °C is not producing resolvable changes to our isotope fractionation model for peridotite melting.

4 Monte Carlo model

We use a Monte Carlo simulation to identify the evolution of pyroxenite behaviour that can best match the calculated $\delta^{57}Fe_{primary}$. For each run of the model, the following steps are taken.

1. The mantle potential temperature at each locality is assigned. The temperature is a random selection within the limits given in Table S2 with the added constraint that the plume must cool through time *(13, 33)*. We note that the mantle potential temperatures used here are generally calculated using major element chemistry (using PRIMELT; *(68)*) without a consideration of lithological heterogeneity (pyroxenite and harzburgite). As shown by Matthews et al. 2021 *(31)*, the consideration of buoyant, low melt-production harzburgite in a plume can reduce the T_p required by olivine chemistry by 100 °C. However, mantle potential temperatures calculated in this way are only available for Tortgual, Gorgona and Curaçao, and therefore for consistency we use potential temperature estimates that have not considered harzburgite in the plume.

2. A source pyroxenite fraction is then randomly chosen for Tortugal and Galápagos, the endmembers of plume evolution in our study. We use the constraint that the plume must be buoyant to set the upper limit of pyroxenite fraction in each case, for the chosen temperature (the lower limit is 0 % pyroxenite). Plume buoyancy is calculated following *(17, 42)*, using the reference ambient mantle density as that of KLB1 peridotite at $T_p = 1300$ °C, and modelling the plume as two lithology mantle (KLB1, and MIX1G – the buoyancy calculations are described in more detail in section S7).

3. A pyroxenite fraction is randomly chosen for each remaining locality, between the endmember Tortugal and Galápagos values, either decreasing or increasing as the plume evolves depending on whether the fraction chosen for Galápagos is higher or lower than that for Tortugal.

4. Given a mantle temperature and pyroxenite fraction for each locality, the aggregate melt δ^{57}Fe can be then calculated, using melt fractions and FeO contents for each locality estimated as outlined in section S3. The aggregate melt δ^{57}Fe was calculated for three different bulk pyroxenite isotope compositions, 0.11, 0.15 and 0.20 ‰.

The simulation ran the model 1000 times, covering the parameter space shown in Fig. S7.

We parameterised the measured $\delta^{57}Fe_{primary}$ at each locality by taking the median of the maximum and minimum values given by the ranges in Fig. 1 of the main text (regardless of primary MgO calculation method), and the difference between these maximum and minimum values. We calculated the misfit for each of the model runs as a chi-square,

$$\chi^2 = \sum \frac{(\delta^{57}Fe_{model} - \delta^{57}Fe_{median\,of\,data})^2}{\sigma^2} \quad (1)$$

where we take σ^2 to be $0.3 \times \delta^{57}Fe_{range\,of\,data}$. 0.3 was chosen as it produces error envelopes on the accepted δ^{57}Fe results that are similar to typical analytical error of the observed data. Changing this value does not affect the minimum misfit results, only the 5th and 95th percentiles of accepted solutions. For example, using a factor

of 0.5 instead of 0.3 and considering the intermediate bulk pyroxenite composition of 0.15 ‰, the 95th percentile of pyroxenite fraction changes from 18–9 % (Tortugal to Galápagos) to 35–9 %.

Taking a 95 % confidence interval we reject any model runs falling above the critical χ^2 value (11.07, one-sided upper tail test). These solutions are shown in Fig. S8, with $<$ 25 % of accepted solutions recording a slightly increasing pyroxenite fraction over time, compared to $>$ 75 % recording a decreasing pyroxenite fraction for the intermediate bulk pyroxenite composition considered. Table S3 shows the results of the minimum misfit solution, and the 5th and 95th percentiles, for each of the bulk isotope compositions considered.

5 Modern day Galápagos heterogeneity

Fig. S9 shows the inter-island variability in δ^{57}Fe in the modern day samples. We also show the results of a sample-by-sample maximum fractional crystallisation correction, as described in *(24)*, using a $\Delta^{57}\mathrm{Fe}_{\mathrm{ol-melt}} = -0.4\times 10^6/\mathrm{T}^2$. Studies looking at source lithology in the modern Galápagos through whole rock analysis (major elements, as well as Fe/Mn ratios) and olivine trace elements show variability across the modern islands, with some volcanoes recording peridotite-dominated source lithologies, and others suggesting up to 20 % pyroxenite in the source, contributing up to 100 % of the melt to some volcanoes *(16, 34)*. However, for the volcanoes studied here, both of these previous studies conclude that peridotite is the dominant melting lithology in the modern plume, consistent with our results of a small pyroxenite fraction (best-fit solutions of 4–12 % pyroxenite across the studied volcanoes). Gleeson et al. *(34)* identify a narrow band of volcanoes that may have a source of up to 20 % pyroxenite, which includes Volcan Wolf, one of our studied volcanoes. This 20 % would be slightly more than our best-fit solutions, but our estimate for the proportion of pyroxenite in the modern Galápagos represents an overall picture of lithology in the western-south western islands, rather than isolating individual volcanoes. This overall picture of lithology seems appropriate given that, although there is radiogenic isotopic variability amongst the islands, both Vidito et al. *(16)* and Gleeson et al. *(34)* conclude that peridotite is the dominant lithology across the plume, with small contributions from pyroxenite, which are only locally dominant (e.g., the island of Santa Cruz). We note that the δ^{57}Fe of the two Volcan Wolf samples measured are individually some of the lowest in the modern dataset, seemingly at odds with its position in the high pyroxenite band *(34)*. However, Vidito et al. *(16)* propose no contribution from pyroxenite at Volcan Wolf based on olivine trace elements, and major element basalt data *(34)* does not clearly tie Volcan Wolf specifically to a high-pyroxenite source lithology.

6 Olivine trace element modelling

We have compared the consistency of our model of approximately constant pyroxenite fraction in the source, as suggested by δ^{57}Fe, with constraints from olivine trace elements. We have calculated the aggregate melt composition for melting of peridotite and bi-lithologic mantle produced by our model at each locality (major element compositions and melt fractions at average pressures of melting calculated/extrapolated as in section

S3). For the bi-lithologic case, the best fit solution of pyroxenite fraction when the bulk pyroxenite composition is 0.15 ‰ was used. The calculations have been done for the range of mantle potential temperatures covered by the model (to highlight the role of temperature on the olivine trace element concentrations in our model). For elements not considered in THERMOCALC outputs, we use the partitioning behaviour during melting and bulk compositions given in Table S4 to calculate the amount of the element in the melt (and other phases present) at the pressures and temperatures of interest. The extrapolation/fitting procedure for high T_p localities from section S3 is then used, as for major elements.

From the major and trace element composition of the aggregate melt calculated for the peridotite-only and bi-lithologic case, we then estimated the equilibrium olivine trace element composition using composition-dependent olivine-melt partition coefficients (Table S5; *(69, 70)*). For any given locality, the range in modelled olivine trace element composition generally depends on two related factors. Firstly, the results depend on the range of potential temperatures proposed for the locality. Secondly, the range in major and trace element composition of an aggregate melt for any locality (hence, the range in modelled olivine trace element composition) depends on the sensitivity of any of the liquid composition parameterisations to that temperature range. For example, the potential temperature range published for Curaçao spans a large change in modelled Ni concentrations in peridotite melts (hence their equilibrium olivine) as the average pressure of melting crosses clinopyroxene-out in the melting assemblage at these temperatures. By contrast, the concentration of Mn in the peridotite melts are not strongly affected by the presence or absence of clinopyroxene (see partition coefficients in Table S4), so the same temperature range does not produce a large range in modelled olivine Mn contents.

In Fig. S10 we compare our model results to published trace element in olivine data (filtered for Fo > 87) for the same localities *(13, 30, 33)*. This data has previously been used to suggest a pyroxenite component only becomes resolvable after the plume head stage *(13, 30, 33)*. We have highlighted the samples in the total olivine dataset that overlap with those used in this study, but note that most samples used here do not have published olivine data. The purpose of our comparison is to show that the results for pyroxenite fraction from Fe isotopes are consistent with the olivine trace element dataset (both for the individual samples used in this study, and the wider olivine trace element data).

We find that some aspects of the natural olivine data can be reproduced by our model of a cooling plume with $<$ 10 % pyroxenite throughout its history. A slight decrease in Ca/Fe in cooler plume tail localities than the plume head, as observed in natural data, can be generated by the plume cooling, regardless of lithology. Our model predicts that in most cases melts from a pyroxenite-bearing source have lower Ca/Fe than pure peridotite melts, in agreement with *(15, 68)*. However, we find that there is minimal difference between the predicted olivine Ca/Fe compositions for the two source compositions (green vs purple bars), and the predicted difference is small compared to the spread of natural data. More extreme variations could be generated by a lower Ca pyroxenite. The trend of decreasing then increasing natural Mn/Fe data as the plume evolves could also be matched by a cooling plume with or without a pyroxenite component; the only data not well-matched in this case is for Gorgona olivines, which have higher Mn/Fe than our pyroxenite-bearing model predicts. We note that, out of the olivine trace element ratios considered here, Mn/Fe appears most sensitive to pyroxenite (with a lower Mn/Fe in olivine

derived from a pyroxenite-bearing source than a peridotite source, consistent with predictions; *(15, 71)*), but that pyroxenite and peridotite Mn concentrations are relatively poorly constrained, which makes modelling their behaviour during bi-lithologic mantle melting relatively uncertain.

The Ni content of olivines has also been proposed as a tracer of pyroxenite, with high Ni indicating pyroxenite in the melt source (e.g., *(15, 72)*). The partitioning of Ni between olivine and melt is dependent on crystallisation temperature as well as melt composition *(70)*, which introduces additional uncertainty into our model where crystallisation temperatures for each locality are poorly constrained. If available, we use crystallisation temperatures from aluminium-in-olivine thermometry (Table S2; *(13)*). The modelled difference in olivine Ni/(Mg/Fe) contents between pure peridotite and bi-lithology source melts is small. This result arises because although the bulk melt-source partition coefficient for olivine-free lithologies (such as MIX1G pyroxenite at depth) is lower than for olivine-bearing peridotite (Ni is compatible in olivine), the pyroxenite has a lower bulk Ni content than peridotite *(15, 71)*, and our model predicts that the Ni contents of the melts from peridotite and a bi-lithologic source will be similar, reflected in the subsequently crystallising olivine. Our model fits the natural olivine data for Tortugal, Gorgona, Curaçao and modern Galápagos, however has a poor fit to Azuero and Quepos, which may be in part due to uncertainties in olivine crystallisation temperature as there are only a few samples with temperature constraints. We also note that, of the samples we measured for δ^{57}Fe that also have olivine separate data, our Tortugal samples sit at the lower end of the natural data range for Ni, and for Quepos at the higher end. However, the olivine composition averages of samples studied for δ^{57}Fe for these two localities are not very different from the average from literature data published by *(33)*, and there are samples measured for δ^{57}Fe that do not have measured olivine compositions. Given the uncertainties in our olivine composition modelling, and the purpose of this figure to highlight that the Fe isotope results are consistent with olivine trace element contents, rather than to draw out specific conclusions about source lithology based only on olivine trace elements, we do not consider the sample distribution for Tortugal and Quepos to be an issue here.

7 Buoyancy and density constraints

The buoyancy of the Galapagos plume, as given in Table S3 for given proportions of pyroxenite, has been calculated following the approach taken by *(17, 42)*. For this study, for consistency across the lithologies used, the subsolidus density of KLB1 and MIX1G with increasing temperature were both calculated using THERMOCALC v. 350 *(58)*, and the latest version of the thermodynamic dataset, ds633 *(56)*, with the relevant activity-composition (a-X) models *(57)*. This THERMOCALC version includes density as a standard output, which was not present in earlier versions of the software. The inputs into THERMOCALC for the two lithologies are given in *(24)*.

Figure 3 in the main text uses geodynamical modelling results from Jones et al. 2019 *(14)* to constrain the density of the lower mantle material carried by the Galápagos plume. In Fig. 3a, we have taken the results from Fig. 2a in *(14)* for the entrainment fraction of dense material in a plume head for two different thicknesses of dense

layer. Buoyancy number, B, is defined here as

$$B = \frac{\Delta\rho_c}{\rho_0 \alpha \Delta T} \tag{2}$$

where $\Delta\rho_c$ is the excess density of the lower mantle material relative to ambient mantle, α is the thermal expansion coefficient, ρ_0 is the reference mantle density and ΔT is the thermal contrast across the boundary layer driving mantle plume development.

In Fig. 3b, we solve equation 2 above to give $(\Delta\rho_c/\rho_0) \times 100$, i.e., the % excess density relative to ambient mantle, for $\alpha - \Delta$T parameter space. The red lines show estimates of excess density given literature estimates of ΔT and α. α applicable to the modelling of the lower mantle is proposed to be between 1×10^{-5} *(9)* and 3×10^{-5} *(14)*. To estimate ΔT ($T_{CMB} - T_p$, following *14*), we use a typical estimate of $T_{CMB} = 3800$ K *(73)* and two estimates of T_p: 2025 K, from our best-fit Monte Carlo T_p estimate for Tortugal for an intermediate pyroxenite bulk composition of 0.15‰; 2425 K, using the Tortugal T_p estimate but considering non-adiabatic cooling processes (e.g., diffusive heat loss) that will mean the T_p estimate in the upper mantle is lower than the plume's T_p at depth. The 400 K estimate of these processes is taken from *(74)* (their Fig. 2).

Supplementary data tables

Data table S1 gives the measured Fe isotope data, along with a compilation of selected literature major and trace element used in this study.

Data table S2 gives the measured Fe isotope data for the geological reference materials used during analytical sessions.

Data table S3 gives the range of calculated primary Fe isotope compositions (as described in this supplement) for each locality.

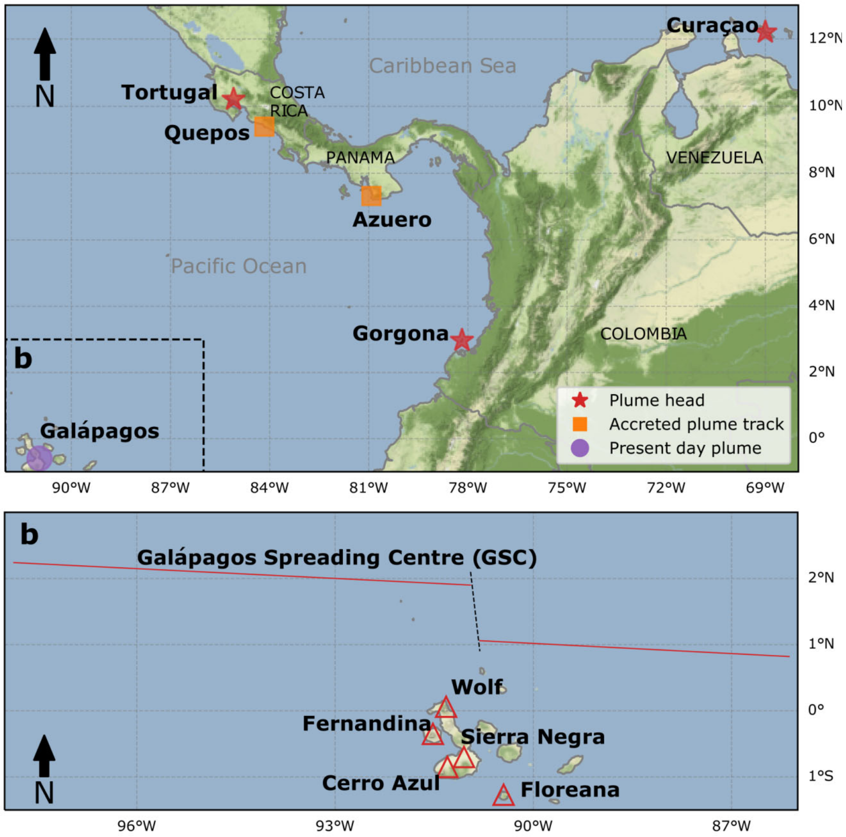

Figure S1: Map of the Galápagos plume-related localities used in this study.

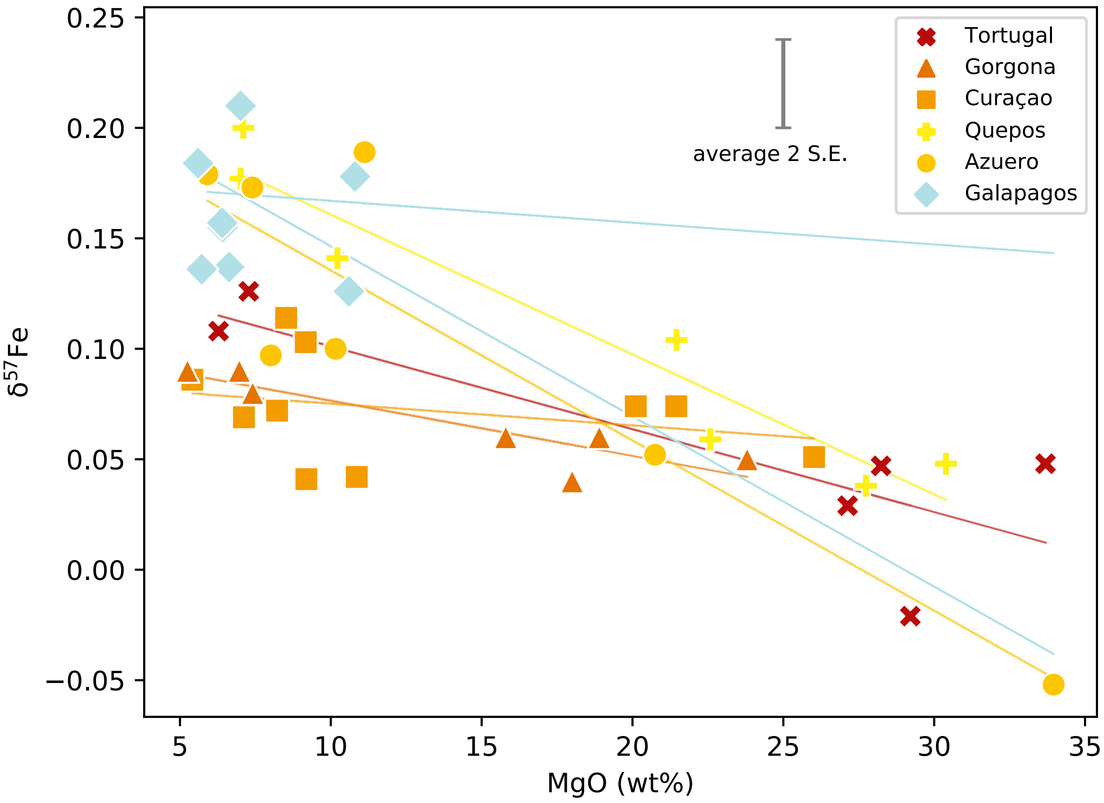

Figure S2: Empirical $\delta^{57}Fe_{measured}$-MgO fits calculated for each locality.

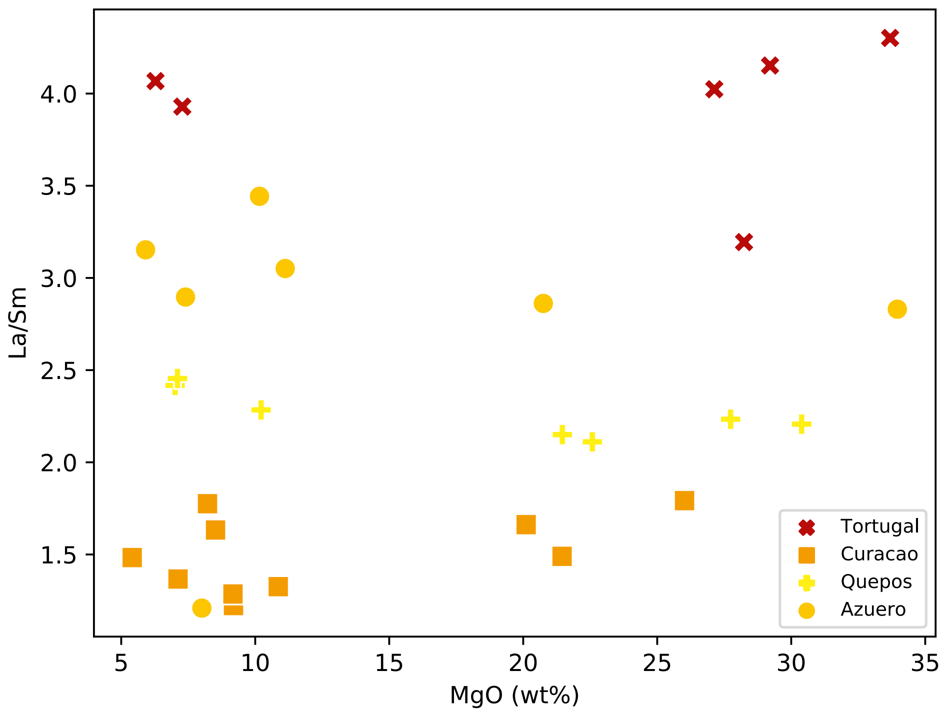

Figure S3: Published La/Sm ratios for the localities where an empirical δ^{57}Fe-MgO fit has been used to correct to $\delta^{57}Fe_{primary}$, to assess the assumption of samples being related by fractional crystallisation/olivine accumulation. No data could be found for the Gorgona samples. Trace element data and sources in Data S1.

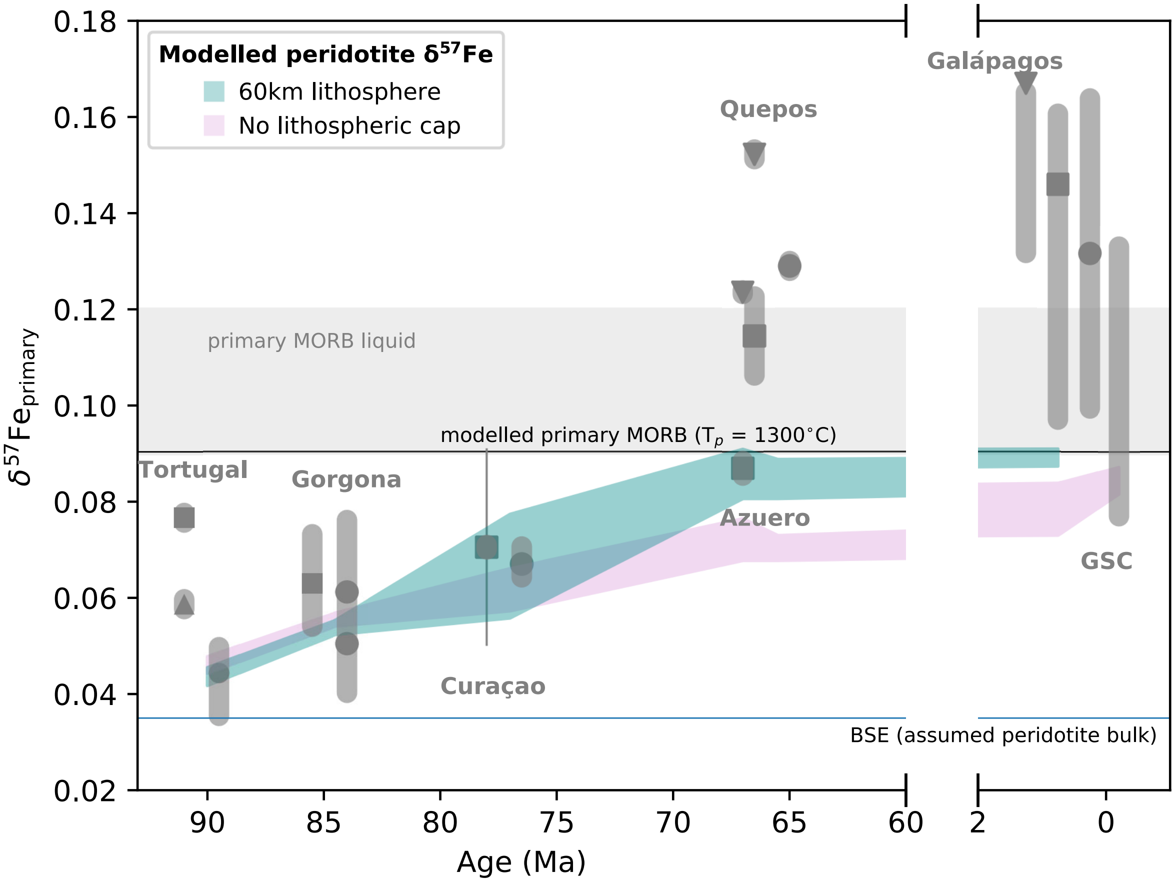

Figure S4: Primary δ^{57}Fe for each locality in the Galápagos plume evolution, compared to modelled peridotite-only melting for a lithospheric thickness of 60 km (for the plume localities) and 0 km (to represent melting at the Galápagos Spreading Centre). Our modelled primary MORB (calculated with 0 km lithospheric thickness and a T_p of 1300 °C) just overlaps with published primary MORB liquid estimates *(24, 39)*.

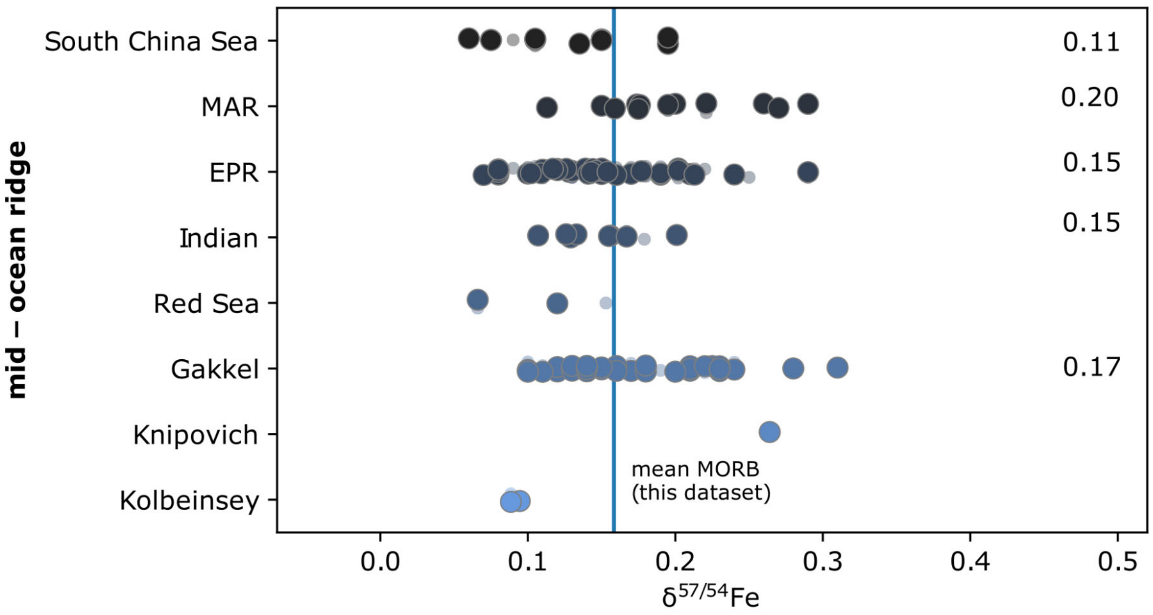

Figure S5: Compilation of published MORB δ^{57}Fe data, separated by ridge segment, to show variation in natural MORB. The numbers on the right-hand side show segment averages (for segments that have sufficient data coverage). Large, outlined circles show samples with 7.2 < MgO (wt%) < 16, to show variability due to fractional crystallisation, but remaining data is shown as pale, smaller circles and all data is used to calculate a segment average. The lowest and highest segment averages (South China Sea, MAR) are used as endmember bulk pyroxenite compositions in our model. Data from *(61–63, 75–78)*.

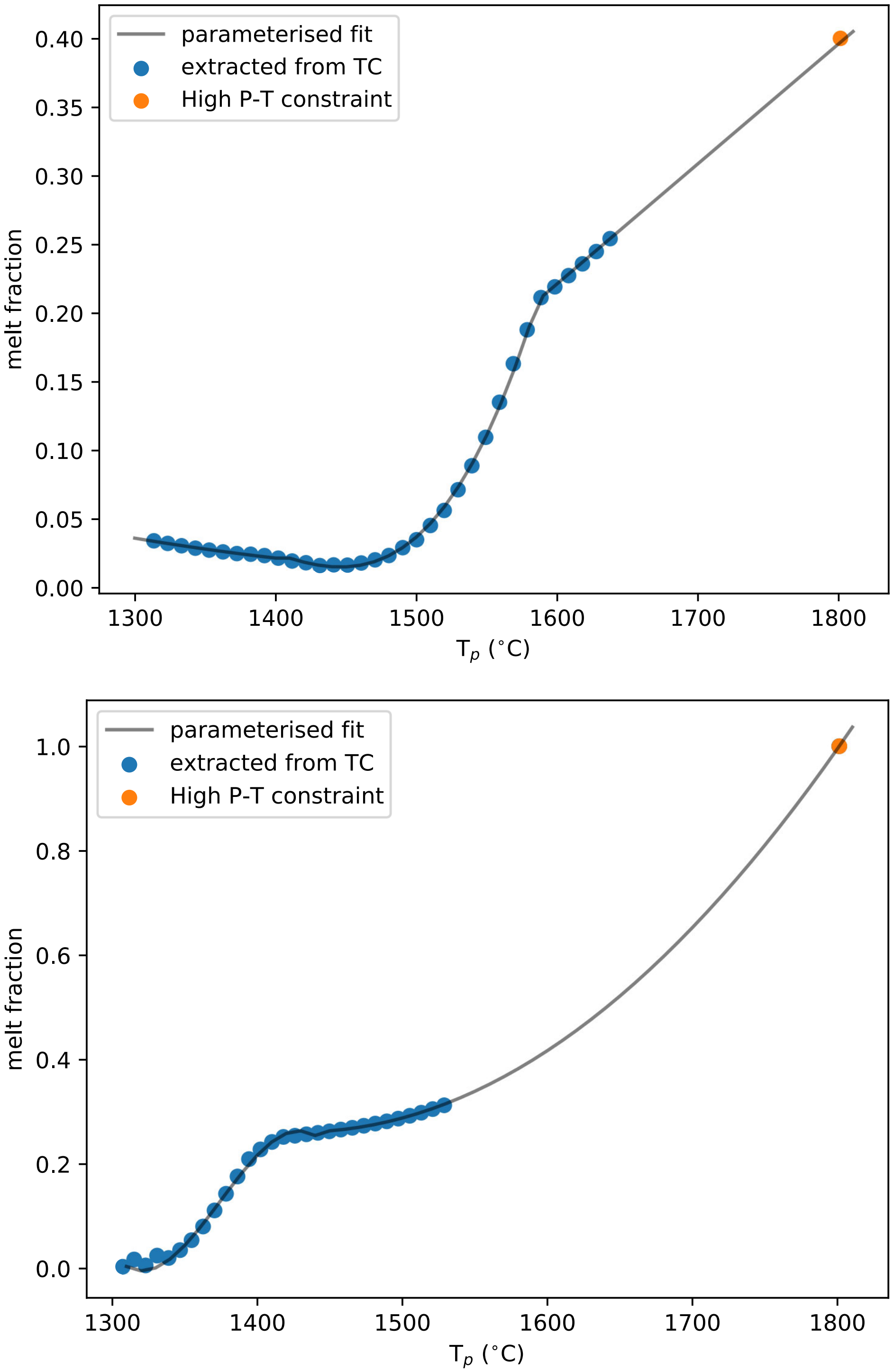

Figure S6: Example of the parameterisation of melt composition and fraction at average pressure of melting (top, for melt fraction of KLB1; bottom, for melt fraction of MIX1G) to higher P-T conditions than the THERMOCALC model. The parameterisation is calculated in terms of mantle potential temperature,T_p, given the assumed linear relationship between average pressure of melting and T_p (°C). The high pressure-temperature experimental constraint for KLB1 (orange point, top panel) is from *(67)*; the calculation of the high pressure-temperature constraint for complete melting of MIX1G is discussed in the text.

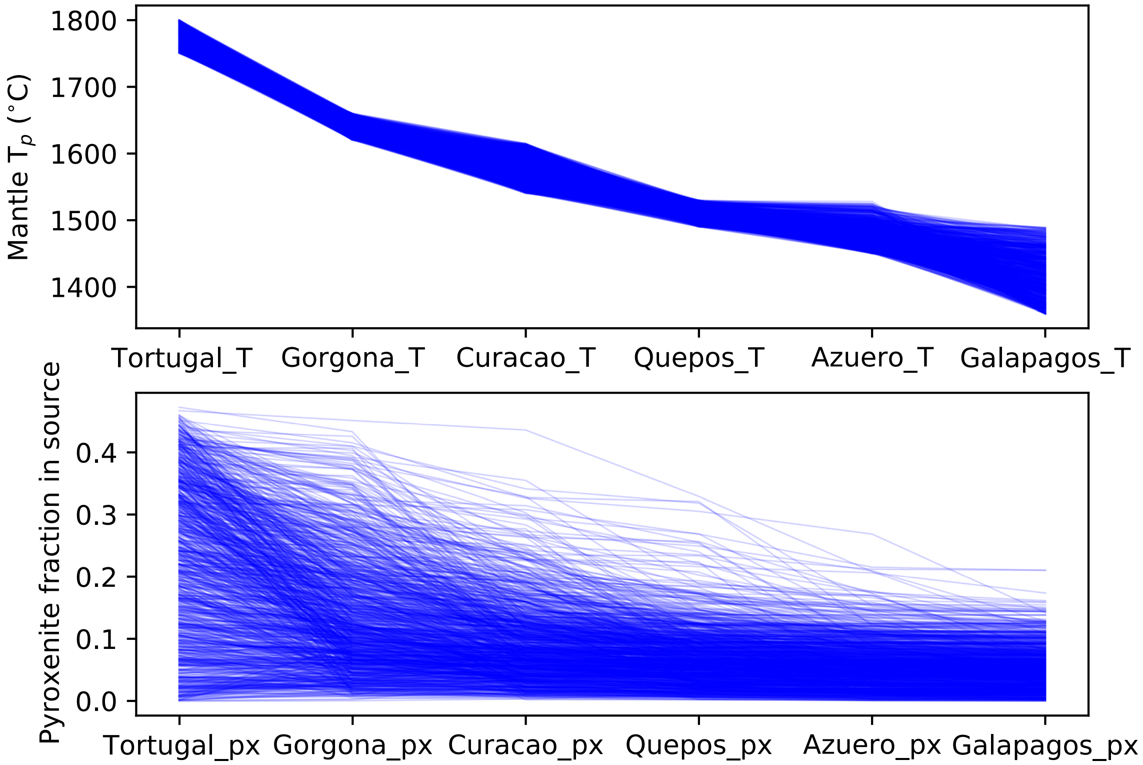

Figure S7: Parameter space covered by the Monte Carlo simulation (1000 runs). Top: mantle T_p, bottom: pyroxenite fraction.

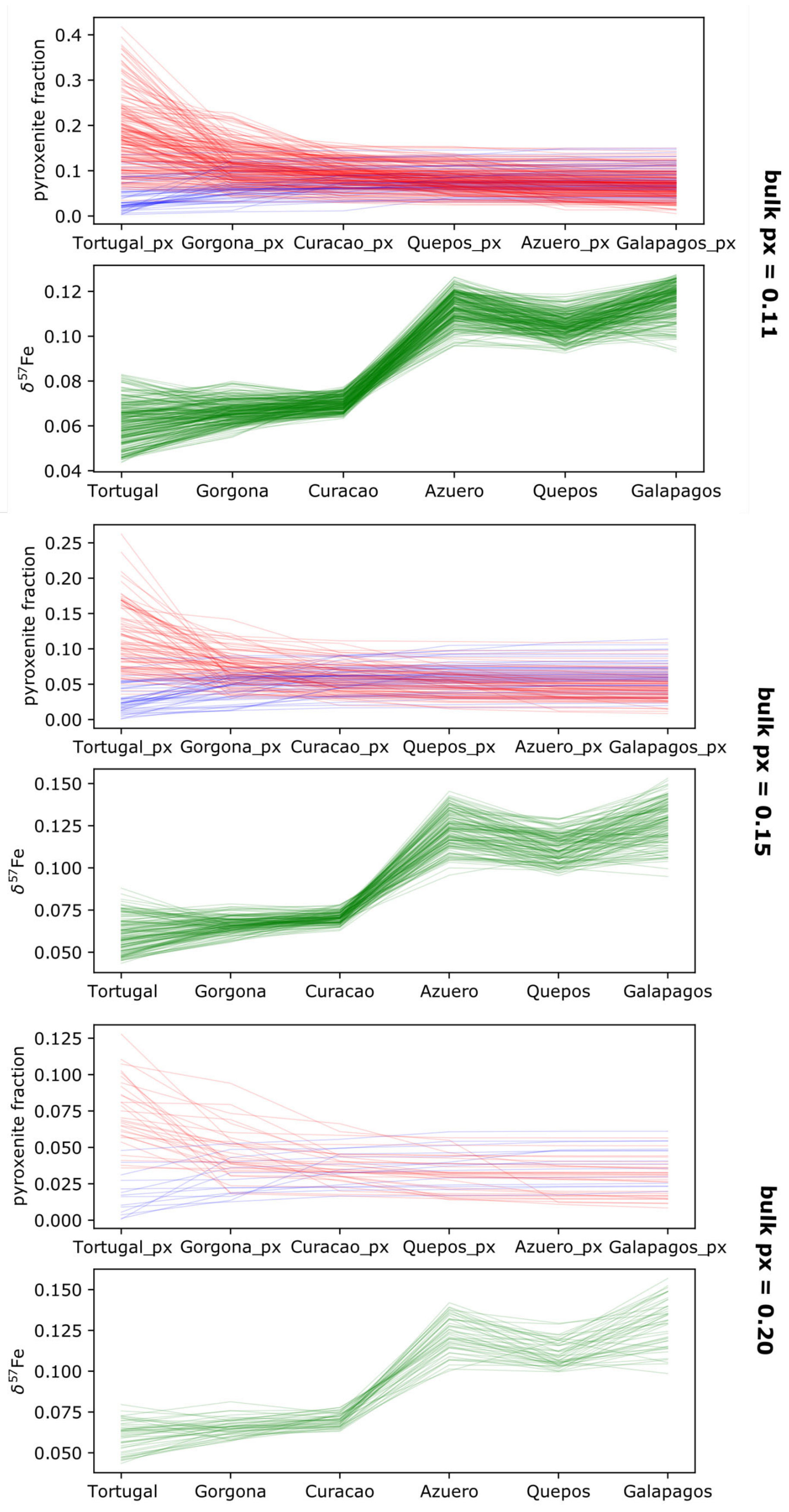

Figure S8: The accepted models from the Monte Carlo simulation, using the critical χ^2 at 95 % confidence, for each of the bulk pyroxenite isotope compositions considered. Top of each pair: evolution of pyroxenite fraction through time, where red solutions are those with decreasing pyroxenite fraction and blue solutions are increasing pyroxenite fraction. Bottom of each pair: δ^{57}Fe for the accepted solutions.

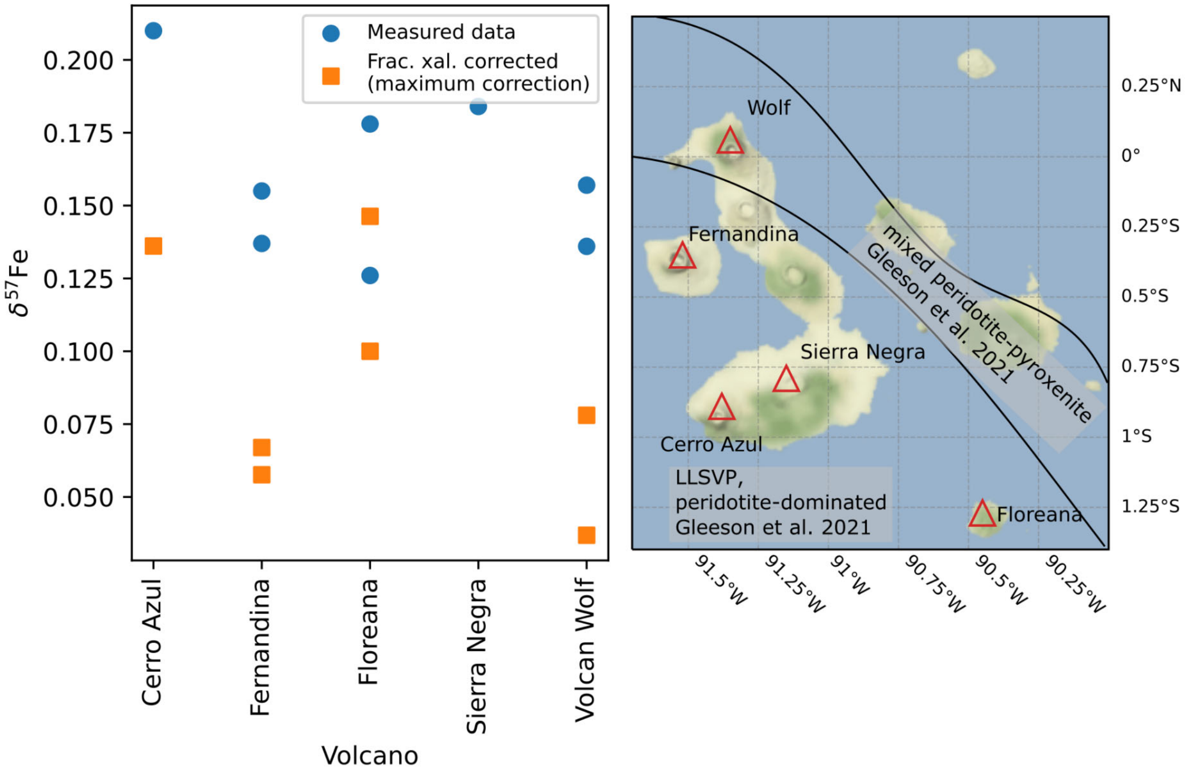

Figure S9: Comparison of Fe isotope data for each of the modern Galápagos volcanoes. Left hand panel compares measured and fractional crystallisation-corrected data (correction as described in text). Right hand panel shows the locations of the modern-day volcanoes studied here compared to the mantle source lithologies proposed by

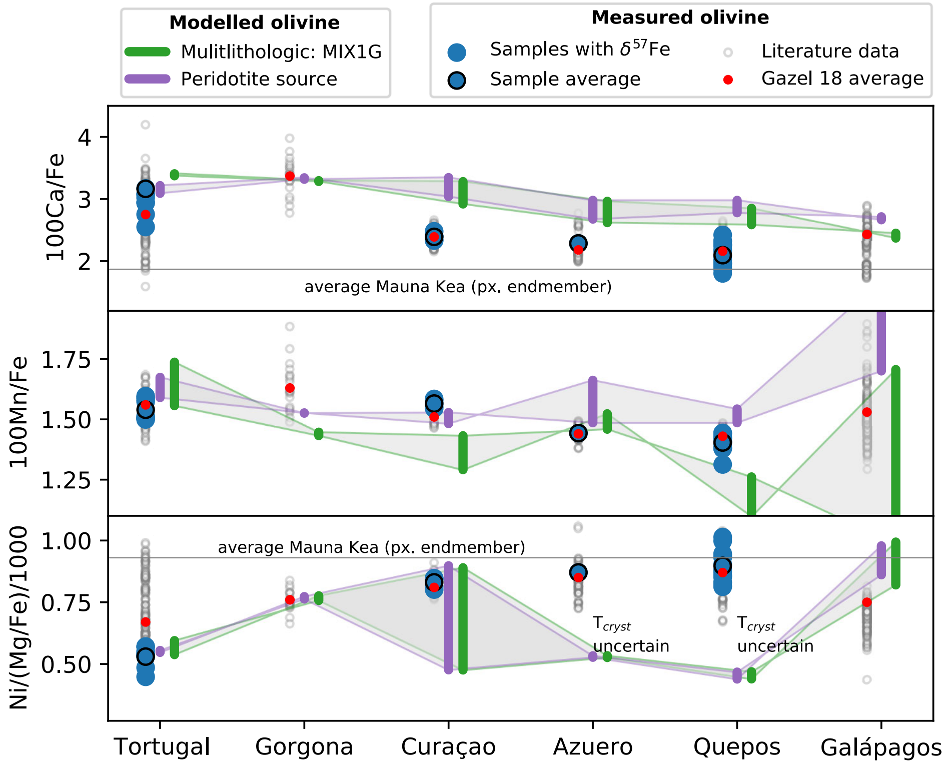

Figure S10: Modelled olivine trace element ratios using the minimum misfit model of small, approximately constant pyroxenite fraction for a bulk pyroxenite composition of 0.15 ‰ (green) and for a pure peridotite source (purple), compared to published olivine trace element data for these localities. See text for details of model parameters. Olivine trace element data from *(13, 30, 33)*, with averages from Gazel et al. 2018 *(33)*. The blue circles highlight the samples in the olivine trace element dataset that have been used in this δ^{57}Fe study. Average Hawaiian Mauna Kea data *(33)* is shown for reference.

Locality	Literature data sources	Literature	MgO wt % range eq. Fo91	eq. Fo88	eq. Fo93
Tortugal	*(79)*		16.5		21.3
	(13)	23.7–27.5			
Gorgona	*(80, 81)*		11.3–19.0		
	(82)	18.0			
	(12)	19.2–22.4			
	(13)	10.1–24.5			
Curaçao	*(83, 84)*		14.2–15.2	10.8	
	(13)	14.6–21.0			
Azuero	*(27, 33, 85)*		16.1–16.5	11.5–11.6	
Quepos	*(79, 86)*		16.0–18.6	11.3	
	(30)	15.0			
Galápagos	*(87)*		16.4–19.7	11.9–14.6	
	(3, 12, 35, 88, 89)	13.2–16.1			

Table S1: The data sources for the major element chemistry required for a calculation of a primary liquid, or estimates of the primary liquid composition. Literature estimates are given next to their reference, all other sources are compiled for the Fo_X calculations.

Locality	T_p max., °C *(source)*	T_p min., °C *(source)*	Age range, Ma *(source)*
Tortugal	1800 *(13)*	1750 *(13)*	95–85 *(13)*$^{+}$
Gorgona	1660 *(13)*	1620 *(12)*	99–70 *(90)**
Curaçao	1615 *(91)*	1540 *(12, 13)*	92–62 *(92)*
Quepos	1530 *(12)*	1490 *(30)*	71–60 *(30, 93)*
Azuero	1530 *(30)*	1450 *(30)*	73–61 *(33)*
Galápagos	1490 *(12)*	1360 *(64)*	1.5–0 *(94)*†

Table S2: Mantle potential temperature limits and locality age ranges used in this study. Where possible, we have used average maximum temperature estimates for the whole locality from each source rather than taking the maximum and minimum on a sample by sample basis. The age range data is given for reference, and is displayed in Fig. 1. $^{+}$Age given as 90 ± 5 Ma. *ages from Gorganilla Island are excluded, since no Gorganilla samples are used in this study. †Floreana is the oldest modern volcano studied.

Locality	T_p (°C)	Solid px. $\%^{\text{95th percentile}}_{\text{5th percentile}}$	Buoyancy (kg m^3)	% Fe from px.	δ^{57}Fe	% W from px.
**Bulk px = 0.11 ‰**						
Tortugal	1771	$11.9\,^{32}_{2}$	−53	21	0.06	89
Gorgona	1646	$12.0\,^{18}_{4}$	−33	20	0.07	85
Curaçao	1612	$12.1\,^{13}_{4}$	−27	20	0.07	85
Quepos	1497	$12.2\,^{13}_{4}$	−9	55	0.12	92
Azuero	1486	$12.2\,^{13}_{3}$	−7	61	0.12	93
Galápagos	1449	$12.2\,^{12}_{3}$	−1	70	0.13	94
**Bulk px = 0.15 ‰**						
Tortugal	1782	$10.2\,^{18}_{1}$	−58	19	0.06	87
Gorgona	1651	$6.3\,^{11}_{3}$	−44	11	0.07	74
Curaçao	1590	$6.3\,^{9}_{3}$	−34	11	0.07	73
Quepos	1510	$6.3\,^{9}_{3}$	−21	31	0.11	82
Azuero	1508	$6.3\,^{9}_{2}$	−21	32	0.11	82
Galápagos	1418	$6.3\,^{9}_{2}$	−6	53	0.14	88
**Bulk px = 0.20 ‰**						
Tortugal	1756	$2.7\,^{11}_{0}$	−67	19	0.05	60
Gorgona	1642	$2.8\,^{7}_{2}$	−48	11	0.06	54
Curaçao	1574	$3.2\,^{6}_{2}$	−37	11	0.07	57
Quepos	1517	$3.7\,^{5}_{2}$	−26	31	0.11	70
Azuero	1512	$4.0\,^{5}_{1}$	−26	32	0.11	73
Galápagos	1437	$4.1\,^{5}_{1}$	−13	53	0.15	84

Table S3: Parameters and results for the minimum misift solution produced by the Monte Carlo simulation, for each bulk isotope composition of pyroxenite considered. px. = pyroxenite.

$D_{min.-melt}$	Ni	Mn	References
Olivine	$\exp(4505/T - 2.075 - \ln(1/D_{MgO}))$	$0.118 + 0.214 \times D_{MgO}$	*(69, 70)*
Garnet	8	1.24 (KLB1), 4.6 (MIX1G)	*(95)*
Opx	3.7	0.64	*(95)*
Cpx	22	0.77 (KLB1), 1.67 (MIX1G)	*(95)*
Spinel	10	0.46	*(95)*
Plag.	0.09	0.03	*(96)*
Perid. bulk	1960 ppm	0.11 (mol % oxide)*	*(97)*
Pyrox. bulk	1000 ppm	0.13 (mol % oxide)	*(59, 72)*

Table S4: Partitioning behaviour and bulk lithology compositions used for elements not calculated by THERMOCALC. *Bulk peridotite Mn is taken to be less than bulk pyroxenite MnO *(98)*, but we note from *(69)* that there is unlikely to be a uniform peridotite trace element composition in the mantle. Cpx = clinopyroxene, opx = orthopyroxene, plag = plagioclase.

Partition coefficient during crystallisation	Calculation
D_{MgO}	$(0.382 - \mathrm{Kd})/0.0164$
D_{FeO}	$\mathrm{Kd} \times D_{MgO}$
D_{CaO}	$-0.019 + 0.007 \times D_{MgO} + 0.063/D_{MgO}$
D_{MnO}	$0.118 + 0.214 \times D_{MgO}$
D_{Ni}	$\exp(4505/T_{cryst.} - 2.075 - \ln(1/D_{MgO}))$

Table S5: Parameterisations used for olivine-melt partition coefficients for low pressure olivine crystallisation. $\mathrm{Kd}_{FeO/MgO} = 0.381 - 0.790/\mathrm{MgO(wt\%)} + 1.039/\mathrm{MgO(wt\%)}^2$; *(69)*. Partitioning behaviour for Mg, Fe, Ca, Mn from *(69)*, for Ni from *(70)*. Average $T_{cryst.}$ estimates used for Ni partitioning are: Tortugal, 1590 °C; Gorgona, 1420 °C; Curaçao, 1350 °C (averages from *(13, 31)*); Azuero & Quepos, ≈ 1300 °C (see *99*); Galápagos, 1130 °C *(100)*.

REFERENCES AND NOTES

1. P. Castillo, The Dupal anomaly as a trace of the upwelling lower mantle. *Nature* **336**, 667–670 (1988).

2. J. Ritsema, S. Ni, D. V. Helmberger, H. P. Crotwell, Evidence for strong shear velocity reductions and velocity gradients in the lower mantle beneath Africa. *Geophys. Res. Lett.* **25**, 4245–4248 (1998).

3. K. S. Harpp, D. Weis, Insights into the origins and compositions of mantle plumes: A comparison of Galápagos and Hawai'i. *Geochem. Geophys. Geosyst.* **21**, e2019GC008887 (2020).

4. M. G. Jackson, J. Blichert-Toft, S. A. Halldórsson, A. Mundl-Petermeier, M. Bizimis, M. D. Kurz, A. A. Price, S. Harðardóttir, L. N. Willhite, K. Breddam, T. W. Becker, R. A. Fischer, Ancient helium and tungsten isotopic signatures preserved in mantle domains least modified by crustal recycling. *Proc. Natl. Acad. Sci. U.S.A.* **117**, 30993–31001 (2020).

5. A. Mundl-Petermeier, R. J. Walker, R. A. Fischer, V. Lekic, M. G. Jackson, M. D. Kurz, Anomalous 182W in high 3He/4He ocean island basalts: Fingerprints of Earth's core? *Geochim. Cosmochim. Acta* **271**, 194–211 (2020).

6. U. R. Christensen, A. W. Hofmann, Segregation of subducted oceanic crust in the convecting mantle. *J. Geophys. Res. Solid Earth* **99**, 19867–19884 (1994).

7. K. Hirose, N. Takafuji, N. Sata, Y. Ohishi, Phase transition and density of subducted MORB crust in the lower mantle. *Earth Planet. Sci. Lett.* **237**, 239–251 (2005).

8. P. J. Tackley, Dynamics and evolution of the deep mantle resulting from thermal, chemical, phase and melting effects. *Earth Sci. Rev.* **110**, 1–25 (2012).

9. E. Mulyukova, B. Steinberger, M. Dabrowski, S. V. Sobolev, Survival of LLSVPs for billions of years in a vigorously convecting mantle: Replenishment and destruction of chemical anomaly. *J. Geophys. Res. Solid Earth* **120**, 3824–3847 (2015).

10. X. Bao, C. R. Lithgow-Bertelloni, M. G. Jackson, B. Romanowicz, On the relative temperatures of Earth's volcanic hotspots and mid-ocean ridges. *Science* **375**, 57–61 (2022).

11. I. H. Campbell, Large igneous provinces and the mantle plume hypothesis. *Elements* **1**, 265–269 (2005).

12. C. Herzberg, E. Gazel, Petrological evidence for secular cooling in mantle plumes. *Nature* **458**, 619–622 (2009).

13. J. Trela, E. Gazel, A. V. Sobolev, L. Moore, M. Bizimis, B. Jicha, V. G. Batanova, The hottest lavas of the Phanerozoic and the survival of deep Archaean reservoirs. *Nat. Geoscience* **10**, 451–456 (2017).

14. T. D. Jones, D. R. Davies, P. A. Sossi, Tungsten isotopes in mantle plumes: Heads it's positive, tails it's negative. *Earth Planet. Sci. Lett.* **506**, 255–267 (2019).

15. A. V. Sobolev, A. W. Hofmann, D. V. Kuzmin, G. M. Yaxley, N. T. Arndt, S.-L. Chung, L. V. Danyushevsky, T. Elliott, F. A. Frey, M. O. Garcia, A. A. Gurenko, V. S. Kamenetsky, A. C. Kerr, N. A. Krivolutskaya, V. V. Matvienkov, I. K. Nikogosian, A. Rocholl, I. A. Sigurdsson, N. M. Sushchevskaya, M. Teklay, The amount of recycled crust in sources of mantle-derived melts. *Science* **316**, 412–417 (2007).

16. C. Vidito, C. Herzberg, E. Gazel, D. J. Geist, K. S. Harpp, Lithological structure of the Galápagos plume. *Geochem. Geophys. Geosyst.* **14**, 4124–4240 (2013).

17. O. Shorttle, J. Maclennan, S. Lambart, Quantifying lithological variability in the mantle. *Earth Planet. Sci. Lett.* **395**, 24–40 (2014).

18. M. L. M. Gleeson, S. A. Gibson, H. M. Williams, Novel insights from Fe-isotopes into the lithological heterogeneity of ocean island basalts and plume-influenced MORBs. *Earth Planet. Sci. Lett.* **535**, 116114 (2020).

19. S.-C. Lin, P. E. van Keken, Dynamics of thermochemical plumes: 1. Plume formation and entrainment of a dense layer. *Geochem. Geophys. Geosyst.* **7**, Q02006 (2006).

20. F. Deschamps, E. Kaminski, P. J. Tackley, A deep mantle origin for the primitive signature of ocean island basalt. *Nat. Geoscience* **4**, 879–882 (2011).

21. M. Li, A. K. McNamara, E. J. Garnero, Chemical complexity of hotspots caused by cycling oceanic crust through mantle reservoirs. *Nat. Geoscience* **7**, 366–370 (2014).

22. C. R. Soderman, O. Shorttle, S. Matthews, H. M. Williams, Global trends in novel stable isotopes in basalts: Theory and observations. *Geochim. Cosmochim. Acta* **318**, 388–414 (2022).

23. O. Nebel, P. A. Sossi, A. Bénard, R. J. Arculus, G. M. Yaxley, J. D. Woodhead, D. R. Davies, S. Ruttor, Reconciling petrological and isotopic mixing mechanisms in the Pitcairn mantle plume using stable Fe isotopes. *Earth Planet. Sci. Lett.* **521**, 60–67 (2019).

24. C. R. Soderman, S. Matthews, O. Shorttle, M. G. Jackson, S. Ruttor, O. Nebel, S. Turner, C. Beier, M.-A. Millet, E. Widom, M. Humayan, H. M. Williams, Heavy δ^{57}Fe in ocean island basalts: A non-unique signature of processes and source lithologies in the mantle. *Geochim. Cosmochim. Acta* **292**, 309–332 (2021).

25. H. M. Williams, M. Bizimis, Iron isotope tracing of mantle heterogeneity within the source regions of oceanic basalts. *Earth Planet. Sci. Lett.* **404**, 396–407 (2014).

26. J. G. Konter, A. J. Pietruszka, B. B. Hanan, V. A. Finlayson, P. R. Craddock, M. G. Jackson, N. Dauphas, Unusual δ^{56}Fe values in Samoan rejuvenated lavas generated in the mantle. *Earth Planet. Sci. Lett.* **450**, 221–232 (2016).

27. K. Hoernle, P. van den Bogaard, R. Werner, B. Lissinna, F. Hauff, G. E. Alvarado, D. Garbe-Schönberg, Missing history (16–71 Ma) of the Galápagos hotspot: Implications for the tectonic and biological evolution of the Americas. *Geology* **30**, 795–798 (2002).

28. P. Denyer, E. Gazel, The Costa Rican Jurassic to Miocene oceanic complexes: Origin, tectonics and relations. *J. South Am. Earth Sci.* **28**, 429–442 (2009).

29. G. E. Alvarado, P. Denyer, C. W. Sinton, The 89 Ma Tortugal komatiitic suite, Costa Rica: Implications for a common geological origin of the Caribbean and Eastern Pacific region from a mantle plume. *Geology* **25**, 439–442 (1997).

30. J. Trela, C. Vidito, E Gazel, C. Herzberg, C. Class, W. Whalen, B. Jicha, M. Bizimis, G. E. Alvarado, Recycled crust in the Galápagos Plume source at 70 Ma: Implications for plume evolution. *Earth Planet. Sci. Lett.* **425,** 268–277 (2015).

31. S. Matthews, K. Wong, O. Shorttle, M. Edmonds, J. Maclennan, Do olivine crystallization temperatures faithfully record mantle temperature variability? *Geochem. Geophys. Geosyst.* **22**, e2020GC009157 (2021).

32. K. Hoernle, R. Werner, J. Phipps Morgan, D. Garbe-Schönberg, J. Bryce, J. Mrazek, Existence of complex spatial zonation in the Galápagos plume. *Geology* **28**, 435–438 (2000).

33. E. Gazel, J. Trela, M. Bizimis, A. V. Sobolev, V. Batanova, C. Class, B. Jicha, Long-lived source heterogeneities in the Galapagos mantle plume. *Geochem. Geophys. Geosyst.* **19**, 2764–2779 (2018).

34. M. L. M. Gleeson, C. R. Soderman, S. Matthews, S. Cottaar, S. Gibson, Geochemical constraints on the structure of the Earth's deep mantle and the origin of the LLSVPs. *Geochem. Geophys. Geosyst.* **22**, e2021GC009932 (2021).

35. K. S. Harpp, P. S. Hall, M. G. Jackson, Galápagos and Easter: A tale of two hotspots, in *The Galapagos: A Natural Laboratory for the Earth Sciences* (Wiley, 2014), vol. 204, chap. 3.

36. X. Ma, X. Sun, C. Thomas, Localized ultra-low velocity zones at the eastern boundary of Pacific LLSVP. *Earth Planet. Sci. Lett.* **507**, 40–49 (2019).

37. M. D. Kurz, J. Curtice, D. Fornari, D. J. Geist, M. Moreira, Primitive neon from the center of the Galápagos hotspot. *Earth Planet. Sci. Lett.* **286**, 23–34 (2009).

38. K. S. Harpp, W. M. White, Tracing a mantle plume: Isotopic and trace element variations of Galápagos seamounts. *Geochem. Geophys. Geosyst.* **2**, e2000GC000137 (2001).

39. P. A. Sossi, O. Nebel, J. Foden, Iron isotope systematics in planetary reservoirs. *Earth Planet. Sci. Lett.* **452**, 295–308 (2016).

40. N. Dauphas, P. R. Craddock, P. D. Asimow, V. C. Bennett, A. P. Nutman, D. Ohnenstetter, Iron isotopes may reveal the redox conditions of mantle melting from Archean to Present. *Earth Planet. Sci. Lett.*, **288**, 255–267 (2009).

41. E. M. Stolper, O. Shorttle, P. M. Antoshechkina, P. D. Asimow, The effects of solid-solid phase equilibria on the oxygen fugacity of the upper mantle. *Am. Min. J. Earth Planet. Mater.* **105**, 1445–1471 (2020).

42. S. Matthews, O. Shorttle, J. Maclennan, The temperature of the Icelandic mantle from olivine-spinel aluminium exchange thermometry. *Geochem. Geophys. Geosyst.*, **17**, 4725–4752 (2016).

43. M. G. Jackson, J. G. Konter, T. W. Becker, Primordial helium entrained by the hottest mantle plumes. *Nature* **542**, 340–343 (2017).

44. A. Ricolleau, J.-P. Perrillat, G. Fiquet, I. Daniel, J. Matas, A. Addad, N. Menguy, H. Cardon, M. Mezouar, N. Guignot, Phase relations and equation of state of a natural MORB: Implications for the density profile of subducted oceanic crust in the Earth's lower mantle. *J. Geophys. Res. Solid Earth* **115,** B08202 (2010).

45. M. G. Jackson, M. D. Kurz, S. R. Hart, R. K. Workman, New Samoan lavas from Ofu Island reveal a hemispherically heterogeneous high 3He/4He mantle. *Earth Planet. Sci. Lett.* **264**, 360–374 (2007).

46. H. M. Williams, A. H. Peslier, C. McCammon, A. N. Halliday, S. Levasseur, N. Teutsch, J.-P. Burg, Systematic iron isotope variations in mantle rocks and minerals: The effects of partial melting and oxygen fugacity. *Earth Planet. Sci. Lett.* **235**, 435–452 (2005).

47. H. M. Williams, S. G. Nielsen, C. Renac, W. L. Griffin, S. Y. O'Reilly, C. A. McCammon, N. Pearson, F. Viljoen, J. C. Alt, A. N. Halliday, Fractionation of oxygen and iron isotopes by partial melting processes: Implications for the interpretation of stable isotope signatures in mafic rocks. *Earth Planet. Sci. Lett.* **283**, 156–166 (2009).

48. P. R. Craddock, N. Dauphas, Iron isotopic compositions of geological reference materials and chondrites. *Geostand. Geoanal. Res.* **35**, 101–123 (2011).

49. F.-Z. Teng, N. Dauphas, R. T. Helz, Iron isotope fractionation during magmatic differentiation in Kilauea Iki lava lake. *Science* **320**, 1620–1622 (2008).

50. A. J. McCoy-West, J. G. Fitton, M.-L. Pons, E. C. Inglis, H. M. Williams, The Fe and Zn isotope composition of deep mantle source regions: Insights from Baffin Island picrites. *Geochim. Cosmochim. Acta* **238**, 542–562 (2018).

51. K. Putirka, Rates and styles of planetary cooling on Earth, Moon, Mars, and Vesta, using new models for oxygen fugacity, ferric-ferrous ratios, olivine-liquid Fe-Mg exchange, and mantle potential temperature. *Am. Mineral.* **101**, 819–840 (2016).

52. E. A. Fisher, K. A. Kelley, M. Brounce, "Investigating plume-influenced mid-ocean ridges: Iron redox conditions and tungsten variation at the Galápagos Spreading Center," (Technical Report No. 13-03, SURFO, 2013), pp. 37–46.

53. E. Cottrell, K. A. Kelley, Redox heterogeneity of the mantle inferred from hotspots, in *AGU Fall Meeting Abstracts*, V23G–02 (2014).

54. H. L. Zhang, E. Cottrell, P. A. Solheid, K. A. Kelley, M. M. Hirschmann, Determination of $Fe^{3+}/\Sigma Fe$ of XANES basaltic glass standards by Mössbauer spectroscopy and its application to the oxidation state of iron in MORB. *Chem. Geol.* **479**, 166–175 (2018).

55. A. Stracke, A process-oriented approach to mantle geochemistry. *Chem. Geol.* **579**, 120350 (2021).

56. T. J. B. Holland, R. Powell, An improved and extended internally consistent thermodynamic dataset for phases of petrological interest, involving a new equation of state for solids. *J. Metam. Geol.* **29**, 333–383 (2011).

57. T. J. B. Holland, E. C. R. Green, R. Powell. Melting of peridotites through to granites: A simple thermodynamic model in the system KNCFMASHTOCr. *J. Petrol.* **59**, 881–900 (2018).

58. R. Powell, T. J. B. Holland, B. Worley, Calculating phase diagrams involving solid solutions via non-linear equations, with examples using THERMOCALC. *J. Metam. Geol.* **16**, 577–588 (1998).

59. S. Lambart, M. B. Baker, E. M. Stolper, The role of pyroxenite in basalt genesis: Melt-PX, a melting parameterization for mantle pyroxenites between 0.9 and 5 GPa *J. Geophys. Res. Solid Earth* **121,** 5708–5735 (2016).

60. C. Johnson, B. Beard, S. Weyer, High-Temperature Fe Isotope Geochemistry, in *Iron Geochemistry: An Isotopic Perspective* (Springer, 2020), chap. 4.

61. O. Nebel, R. J. Arculus, P. A. Sossi, F. E. Jenner, T. H. E. Whan, Iron isotopic evidence for convective resurfacing of recycled arc-front mantle beneath back-arc basins. *Geophys. Res. Lett.* **40** (22), 5849–5853 (2013).

62. F.-Z. Teng, N. Dauphas, S. Huang, B. Marty, Iron isotopic systematics of oceanic basalts. *Geochim. Cosmochim. Acta* **107**, 12–26 (2013).

63. Y. Zhong, G.-L. Zhang, W.-X. Lv, F. Huang, Iron isotope constraints on the lithological heterogeneity of the upper mantle in the South China Sea. *J. Asian Earth Sci.* **220**, **104934** (2021).

64. S. A. Gibson, D. J. Geist, Geochemical and geophysical estimates of lithospheric thickness variation beneath Galápagos. *Earth Planet. Sci. Lett.* **300**, 275–286 (2010).

65. S. Révillon, N. T. Arndt, E. Hallot, A. C. Kerr, J. Tarney, Petrogenesis of picrites from the Caribbean Plateau and the North Atlantic magmatic province. *Lithos* **49**, 1–21 (1999).

66. S. Lambart, D. Laporte, P. Schiano, Markers of the pyroxenite contribution in the major-element compositions of oceanic basalts: Review of the experimental constraints. *Lithos* **160–161**, 14–36 (2013).

67. E. Takahashi, T. Shimazaki, Y. Tsuzaki, H. Yoshida, Melting study of a peridotite KLB-1 to 6.5 GPa, and the origin of basaltic magmas. *Philos. Trans. Royal Soc.* **342**, 105–120 (1993).

68. C. Herzberg, M. J. O'Hara, Plume-associated ultramafic magmas of Phanerozoic age. *J. Petrol.* **43**, 1857–1883 (2002).

69. A. K. Matzen, M. B. Baker, J. R. Beckett, B. J. Wood, E. M. Stolper, The effect of liquid composition on the partitioning of Ni between olivine and silicate melt. *Contrib. Mineral. Petrol.* **172**, 3 (2017).

70. C. Herzberg, P. D. Asimow, Petrology of some oceanic island basalts: PRIMELT2. XLS software for primary magma calculation. *Geochem. Geophys. Geosyst.* **9**, 2008GC002057 (2008).

71. C. Herzberg, Identification of source lithology in the Hawaiian and Canary Islands: Implications for origins. *J. Petrol.* **52**, 113–146 (2011).

72. A. V. Sobolev, A. W. Hofmann, S. V. Sobolev, I. K. Nikogosian, An olivine-free mantle source of Hawaiian shield basalts. *Nature* **434**, 590–597 (2005).

73. F. Deschamps, Y. Li, P. J. Tackley, Large-scale thermo-chemical structure of the deep mantle: observations and models, in *The Earth's heterogeneous mantle* (Springer, 2015), pp. 479–515.

74. M. Albers, U. R. Christensen, The excess temperature of plumes rising from the core-mantle boundary. *Geophys. Res. Lett.* **23**, 3567–3570 (1996).

75. S. Weyer, D. A. Ionov, Partial melting and melt percolation in the mantle: The message from Fe isotopes. *Earth Planet. Sci. Lett.* **259**, 119–133 (2007).

76. S. Chen, Y. Niu, P. Guo, H. Gong, P. Sun, Q. Xue, M. Duan, X. Wang, Iron isotope fractionation during mid-ocean ridge basalt (MORB) evolution: Evidence from lavas on the East Pacific Rise at 10°30' N and its implications. *Geochim. Cosmochim. Acta* **267**, 227–239 (2019).

77. P. Sun, Y. Niu, P. Guo, M. Duan, S. Chen, H. Gong, X. Wang, Y. Xiao, Large iron isotope variation in the eastern Pacific mantle as a consequence of ancient low-degree melt metasomatism. *Geochim. Cosmochim. Acta* **286**, 269–288 (2020).

78. M. Richter, O. Nebel, M. Schwindinger, Y. Nebel-Jacobsen, H. J. B. Dick, Competing effects of spreading rate, crystal fractionation and source variability on Fe isotope systematics in mid-ocean ridge lavas. *Sci. Rep.* **11**, 4123 (2021).

79. F. Hauff, K. Hoernle, P. van den Bogaard, G. Alvarado, D. Garbe-Schönberg, Age and geochemistry of basaltic complexes in western Costa Rica: Contributions to the geotectonic evolution of Central America. *Geochem. Geophys. Geosyst.* **1**, 1999GC000020 (2000b).

80. B. Dupré, L. M. Echeverria, Pb isotopes of Gorgona Island (Colombia): Isotopic variations correlated with magma type. *Earth Planet. Sci. Lett.* **67**, 186–190 (1984).

81. R. J. Walker, L. M. Echeverria, S. B. Shirey, M. F. Horan, Re—Os isotopic constraints on the origin of volcanic rocks, Gorgona Island, Colombia: Os isotopic evidence for ancient heterogeneities in the mantle. *Contrib. Mineral. Petrol.* **107**, 150–162 (1991).

82. A. C. Kerr, G. F. Marriner, N. T. Arndt, J. Tarney, A. Nivia, A. D. Saunders, R. A. Duncan, The petrogenesis of Gorgona komatiites, picrites and basalts: New field, petrographic and geochemical constraints. *Lithos* **37**, 245–260 (1996b).

83. A. C. Kerr, J. Tarney, G. F. Marriner, G. T. Klaver, A. D. Saunders, M. F. Thirlwall, The geochemistry and petrogenesis of the late-Cretaceous picrites and basalts of Curaçao, Netherlands Antilles: A remnant of an oceanic plateau. *Contrib. Mineral. Petrol.* **124**, 29–43 (1996).

84. F. Hauff, K. Hoernle, G. Tilton, D. W. Graham, A. C. Kerr, Large volume recycling of oceanic lithosphere over short time scales: Geochemical constraints from the Caribbean large igneous province. *Earth Planet. Sci. Lett.* **174**, 247–263 (2000).

85. W. Wegner, G. Wörner, R. S. Harmon, B. R. Jicha, Magmatic history and evolution of the Central American Land Bridge in Panama since Cretaceous times. *GSA Bulletin* **123**, 703–724 (2011).

86. W. Frisch, M. Meschede, M. Sick, Origin of the Central American ophiolites: Evidence from paleomagnetic results. *Geol. Soc. Am. Bull.* **104**, 1301–1314 (1992).

87. T. Naumann, D. J. Geist, M. Kurz, Petrology and geochemistry of Volcan Cerro Azul: Petrologic diversity among the western Galapagos volcanoes. *J. Petrol.* **43**, 859–883 (2002).

88. D. J. Geist, T. R. Naumann, J. J. Standish, M. D. Kurz, K. S. Harpp, W. M. White, D. J. Fornari, Wolf Volcano, Galápagos Archipelago: Melting and magmatic evolution at the margins of a mantle plume. *J. Petrol.* **46**, 2197–2224 (2005).

89. H. K. Handley, S. Turner, K. Berlo, C. Beier, A. E. Saal, Insights into the Galápagos plume from uranium-series isotopes of recently erupted basalts. *Geochem. Geophys. Geosyst.* **12**, 2011GC003676 (2011).

90. L. Serrano, L. Ferrari, M. L. Martínez, C. M. Petrone, C. Jaramillo, An integrative geologic, geochronologic and geochemical study of Gorgona Island, Colombia: Implications for the formation of the Caribbean Large Igneous Province. *Earth Planet. Sci. Lett.* **309**, 324–336 (2011).

91. A. R. Hastie, A. C. Kerr, Mantle plume or slab window? Physical and geochemical constraints on the origin of the Caribbean oceanic plateau. *Earth Sci. Rev.* **98**, 283–293 (2010).

92. M. W. Loewen, R. A. Duncan, A. J. R. Kent, K. Krawl, Prolonged plume volcanism in the Caribbean Large Igneous Province: New insights from Curaçao and Haiti. *Geochem. Geophys. Geosyst.* **14**, 4241–4259 (2013).

93. C. W. Sinton, R. A. Duncan, P. Denyer, Nicoya Peninsula, Costa Rica: A single suite of Caribbean oceanic plateau magmas. *J. Geophys. Res. Solid Earth* **102**, 15507–15520 (1997).

94. K. S. Harpp, D. J. Geist, A. M. Koleszar, B. Christensen, J. Lyons, M. Sabga, N. Rollins, The geology and geochemistry of Isla Floreana, Galápagos: A different type of late-stage ocean island volcanism, in *The Galápagos: A Natural Laboratory for the Earth Sciences* (American Geophysical Union, 2014).

95. V. Le Roux, R. Dasgupta, C.-T. A. Lee, Recommended mineral-melt partition coefficients for FRTEs (Cu), Ga, and Ge during mantle melting. *Am. Mineral.* **100**, 2533–2544 (2015).

96. M. Laubier, T. L. Grove, C. H. Langmuir, Trace element mineral/melt partitioning for basaltic and basaltic andesitic melts: An experimental and laser ICP-MS study with application to the oxidation state of mantle source regions. *Earth Planet. Sci. Lett.* **392**, 265–278 (2014).

97. V. J. M. Salters, A. Stracke, Composition of the depleted mantle. *Geochem. Geophys. Geosyst.* **5**, 2003GC000597 (2004).

98. F. A. Davis, J. A. Tangeman, T. J. Tenner, M. M. Hirschmann, The composition of KLB-1 peridotite. *Am. Mineral.* **94**, 176–180 (2009).

99. E. Gazel, A. V. Sobolev, M. Bizimis, C. Class, B. R. Jicha, Long-lived source heterogeneities in the Galápagos mantle plume, in *Proceedings of the AGU Fall Meeting Abstracts*, V23H–0206 (2019).

100. M. J. Stock, D. J. Geist, D. A. Neave, M. L. M. Gleeson, B. Bernard, K. A. Howard, I. Buisman, J. Maclennan, Cryptic evolved melts beneath monotonous basaltic shield volcanoes in the Galápagos Archipelago. *Nat. Commun.* **11**, 1–13 (2020).
